# Whole-genome sequence-based genomic prediction in laying chickens with different genomic relationship matrices to account for genetic architecture

**DOI:** 10.1186/s12711-016-0277-y

**Published:** 2017-01-16

**Authors:** Guiyan Ni, David Cavero, Anna Fangmann, Malena Erbe, Henner Simianer

**Affiliations:** 1 Animal Breeding and Genetics Group, Georg-August-Universität, Göttingen, Germany; 2 Lohmann Tierzucht GmbH, Cuxhaven, Germany; 3 Institute for Animal Breeding, Bavarian State Research Centre for Agriculture, Grub, Germany

## Abstract

**Background:**

With the availability of next-generation sequencing technologies, genomic prediction based on whole-genome sequencing (WGS) data is now feasible in animal breeding schemes and was expected to lead to higher predictive ability, since such data may contain all genomic variants including causal mutations. Our objective was to compare prediction ability with high-density (HD) array data and WGS data in a commercial brown layer line with genomic best linear unbiased prediction (GBLUP) models using various approaches to weight single nucleotide polymorphisms (SNPs).

**Methods:**

A total of 892 chickens from a commercial brown layer line were genotyped with 336 K segregating SNPs (array data) that included 157 K genic SNPs (i.e. SNPs in or around a gene). For these individuals, genome-wide sequence information was imputed based on data from re-sequencing runs of 25 individuals, leading to 5.2 million (M) imputed SNPs (WGS data), including 2.6 M genic SNPs. De-regressed proofs (DRP) for eggshell strength, feed intake and laying rate were used as quasi-phenotypic data in genomic prediction analyses. Four weighting factors for building a trait-specific genomic relationship matrix were investigated: identical weights, −(log_10_ *P*) from genome-wide association study results, squares of SNP effects from random regression BLUP, and variable selection based weights (known as BLUP|GA). Predictive ability was measured as the correlation between DRP and direct genomic breeding values in five replications of a fivefold cross-validation.

**Results:**

Averaged over the three traits, the highest predictive ability (0.366 ± 0.075) was obtained when only genic SNPs from WGS data were used. Predictive abilities with genic SNPs and all SNPs from HD array data were 0.361 ± 0.072 and 0.353 ± 0.074, respectively. Prediction with −(log_10_ *P*) or squares of SNP effects as weighting factors for building a genomic relationship matrix or BLUP|GA did not increase accuracy, compared to that with identical weights, regardless of the SNP set used.

**Conclusions:**

Our results show that little or no benefit was gained when using all imputed WGS data to perform genomic prediction compared to using HD array data regardless of the weighting factors tested. However, using only genic SNPs from WGS data had a positive effect on prediction ability.

**Electronic supplementary material:**

The online version of this article (doi:10.1186/s12711-016-0277-y) contains supplementary material, which is available to authorized users.

## Background

Genomic prediction (GP) uses genomic information to obtain estimated breeding values, which are subsequently used to select candidate individuals [1]. GP has been widely implemented in livestock [2–4] and plant [5] breeding schemes. The availability of next-generation sequencing technologies has made it possible to apply GP with whole-genome sequencing (WGS) data. GP with WGS is expected to lead to higher predictive ability, since WGS data include a large number of genomic variants including most of the causal mutations. Thus, prediction depends much less on linkage disequilibrium (LD) between single nucleotide polymorphisms (SNPs) and causal mutations. Furthermore, Georges [6] claimed that WGS data can measure segregation of SNPs properly, which is not the case of commercial chips, particularly for rare SNPs. Based on a simulation study, Pérez-Enciso et al. [7] stated that using WGS data did not increase prediction accuracy compared to high-density (HD) array data. In a first study using sequenced inbred lines of *Drosophila melanogaster*, prediction based on WGS data using ~2.5 million (M) SNPs did not increase accuracy compared to an approach using only ~5% of the segregating SNPs [8]. In cattle data, Hayes et al. [9] found that accuracy of GP was improved by only 2% with WGS data compared to the 800 K array data when using BayesRC and imputed 1000 Bull genomes data. In addition, Van Binsbergen et al. [10] reported that GP with imputed WGS data did not lead to a higher prediction accuracy, compared to the HD array data from more than 5000 Holstein–Friesian bulls. Brøndum et al. [11] showed that the reliability of GP could be improved by adding several significant quantitative trait loci (QTL), which were detected by genome-wide association studies (GWAS) of WGS data, to the regular 54 K bovine array data, especially for production traits. Thus, GP with WGS data could be attractive, although so far the expectations for higher accuracies have not been realized with real data on cattle.

In chicken, most previous studies regarding GP were based on commercial array data. For instance, Morota et al. [12] reported that GP accuracy was higher when using all available SNPs than when using only validated SNPs from a partial genome (e.g. coding regions), based on the 600 K SNP array data of 1351 commercial broiler chicken. Abdollahi-Arpanahi et al. [13] studied 1331 chicken which were genotyped with a 600 K Affymetrix platform and phenotyped for body weight; they reported that predictive ability increased by adding the top 20 SNPs with the largest effects that were detected in the GWAS as fixed effects in the genomic best linear unbiased prediction (GBLUP) model. So far, studies to evaluate the predictive ability with WGS data in chicken are rare. Heidaritabar et al. [14] studied imputed WGS data from 1244 white layer chickens, which were imputed from 60 K SNPs up to sequence level with 22 sequenced individuals as reference samples. They reported a small increase (~1%) in predictive ability for the trait ‘number of eggs’ by using WGS data compared to 60 K SNPs when using a GBLUP model, while there was no difference when using a BayesC model.

Regardless of the genotyping source (i.e. WGS data or array data) used, GBLUP has been widely used in GP studies. Besides GBLUP in its classical form, in which each SNP is assumed to have the same contribution to the genetic variance, several weighting factors for SNPs or parts of the SNP set were proposed to account for the genetic architecture [15–17]. De los Campos et al. [15] proposed a method using the −(log_10_ *P*) from GWAS as a weighting factor for each SNP to build a genomic relationship matrix (**G** matrix). They observed that prediction accuracy for human height was improved compared to the original GBLUP, based on ~6000 records that were drawn from a public human type-2 diabetes case–control dataset with a 500 K SNP platform. Zhou et al. [16] used LD phase consistency, or estimated SNP effects or both as weighting factors to build a weighted **G** matrix, and reported that GBLUP with those weighted **G** matrices did not lead to higher GP accuracy in a study based on 5215 Nordic Holstein bulls and 4361 Nordic Red bulls. Using a German Holstein dataset, Zhang et al. [17] reported that the performance of BLUP given genomic architecture (BLUP|GA), which puts an optimal weight on a subset of SNPs with the strongest effects from the training set was similar to that of GBLUP for somatic cell score (SCS), but that BLUP|GA outperformed GBLUP for fat percentage and milk yield. The advantages of BLUP|GA were larger when the datasets were relatively small.

The objective of this study was to compare results from genomic prediction analyses using both HD array data and WGS data that were performed with GBLUP models and a variety of weighting factors for specific SNPs in a purebred commercial brown layer chicken line.

## Methods

### Data

#### High-density array data

We used 892 female and male chickens from six generations from a purebred commercial brown layer line (see Additional file 1: Table S1 for the number of individuals in each generation). These chickens were genotyped with the Affymetrix Axiom^®^ Chicken Genotyping Array (denoted as the HD array), which initially included 580 K SNPs. Genotype data were pruned by removing SNPs located on the sex chromosomes and in unmapped linkage groups, and SNPs with a minor allele frequency (MAF) lower than 0.5% or a genotyping call rate lower than 97%. Individuals with call rates lower than 95% were also discarded. After filtering, 336,224 SNPs that segregated for 892 individuals remained for analyses.

#### Imputed whole-genome sequence data

Data from re-sequencing that were obtained with the Illumina HiSeq2000 technology with a target coverage of 8× were available for 25 brown layer chickens of the same population (of which 18 were also genotyped with the HD array) and for another 25 white layer chickens. Chickens used for whole-genome sequencing were chosen from the older generations and with a maximum relationship with the chickens that were to be imputed [18, 19]. Data from re-sequencing runs (brown and white layer chickens) were aligned to Build 4 of the chicken reference genome (galGal4) with BWA (version 0.7.9a-r786) [20] using default parameters for paired-end alignment and SNP variants were called using GATK (version 3.1-1-g07a4bf8, UnifiedGenotyper) [21]. Called variants (only for the 25 brown layers) were edited for depth of coverage (DP) and mapping quality (MQ) based on the following criteria: (1) for DP, outlier SNPs (at the top 0.5% of DP) were removed, then, mean and standard deviations of DP were calculated for the remaining SNPs and those that had a DP above and below 3 times the standard deviation from the mean were removed; and (2) for MQ, SNPs with a MQ lower than 30 (corresponding to a probability of 0.001 that their position on the genome was not correct) were removed. After filtering, within the set of 25 re-sequenced brown layers, 10,420,560 SNPs remained and were used as the reference dataset to impute HD array data up to sequence level. Imputation of all genotyped individuals was then performed using Minimac3 [22] which needs pre-phased data as input. The pre-phasing procedure was done with the BEAGLE 4 package [23]. Default numbers of iteration were used in pre-phasing and imputation. The imputation process did not use pedigree information. According to our previous study [24], phasing genotype data with BEAGLE 4 and further imputing with Minimac3 provided the highest imputation accuracy under different validation strategies. After imputation, post-imputation filtering criteria were applied per SNP, namely, SNPs with a MAF lower than 0.5% or SNPs with an imputation accuracy lower than 0.8 were removed. The imputation accuracy used here was the Rsq measurement from Minimac3, which was the estimated value of the squared correlation between true and imputed genotypes. After this step, 5,243,860 imputed SNPs were available for 892 individuals, which are hereafter denoted as WGS data.

In addition, SNPs, regardless of which dataset they were in, were classified into nine classes by gene-based annotation with the ANNOVAR software [25] by setting default parameters and using galGal4 as reference genome [26]. Our set of genic SNPs (SNP_genic) included all SNPs from the eight categories exon, splicing, ncRNA, UTR5′, UTR3′, intron, upstream, and downstream regions of the genome, whereas the ninth category included SNPs from intergenic regions. There were 2,593,054 SNPs characterized as genic SNPs from the WGS data (hereafter denoted as WGS_genic data) and 157,393 SNPs characterized as genic SNPs from the HD array data (hereafter denoted as HD_genic data).

#### Phenotypic observations

The quasi-phenotypic data were de-regressed proofs (DRP) for eggshell strength (ES), feed intake (FI), and arcsine transformed laying rate in the last third of the laying period (LR). The arcsine transformation of the latter trait was performed to achieve an approximate normalization. To obtain de-regressed proofs, a single trait BLUP animal model was performed for each trait using raw phenotypic and pedigree data, respectively. Estimated breeding values from these models were then de-regressed following Garrick et al. [27]. The de-regression process included removal of the parent average information.

### Genomic prediction

Genomic prediction was performed using the following GBLUP model with different genomic relationship matrices that are described below:

$${\mathbf{y}} = {\mathbf{X}}\mu + {\mathbf{Zg}} + {\mathbf{e}},$$

where **y** is the vector of DRP of individuals in the training set for a specific trait; *μ* is the overall mean; **g** is the vector of additive genetic values (i.e. genomic breeding values) for all genotyped chickens; **e** is the vector of residual terms; **X** and **Z** are design matrices assigning DRP to the overall mean and additive genetic values, where the dimension of **Z** is the number of individuals in the training set times the number of all genotyped individuals.

A normal distribution of the residual term **e** is assumed ${\mathbf{e}}\sim{{N}}\left( {0,{\mathbf{R}}\sigma_{e}^{2} } \right)$, where **R** is a diagonal matrix, with diagonal element $R_{ii} = \left( {1 - r_{DRPi}^{2} } \right)/r_{DRPi}^{2}$ [28] for an individual *i* in the training set, where $r_{DRPi}^{2}$ is the reliability of DRP for individual *i*, and $\sigma_{e}^{2}$ is the residual variance. The distribution of the additive genetic values is assumed normal ${\mathbf{g}}\sim{{N}}\left( {0,{\mathbf{G}}\sigma_{g}^{2} } \right)$, where $\sigma_{g}^{2}$ is the additive genetic variance and **G** is a realized genomic relationship matrix including all genotyped individuals, which can be calculated with different approaches resulting in different GBLUP models.

The general approach to build a **G** matrix is:

$${\mathbf{G}} = \frac{{{\mathbf{MDM}}^{{\mathbf{T}}} }}{{2\sum\nolimits_{i = 1}^{m} {p_{i} \left( {1 - p_{i} } \right)} }},$$

where **M** contains the corrected SNP genotypes with individuals in rows and SNPs in columns. The elements of column *i* of **M** are 0 − 2*p*_*i*_ (for homozygotes of the first allele), 1 − 2*p*_*i*_ (for heterozygotes), and 2 − 2*p*_*i*_ (for homozygotes of the second allele), where *p*_*i*_ is the frequency of the second allele at locus *i* from the current dataset. **D** is a diagonal matrix that contains the weight of each locus; these weights varied according to the scenario studied. An identity matrix was used (**D** = **I**) in the original GBLUP [29], which implies that all loci contribute equally to the variance–covariance structure. The resulting **G** matrix is denoted as **G**_**I**_ in the following. De los Campos et al. [15] suggested using the corresponding −(log_10_ *P*) from a *t* test of a GWAS as weighting factors to consider the relative importance of different SNPs on a specific trait. The genomic relationship matrix including a **D** matrix based on this weighing factor will be denoted as **G**_**P**_. The corresponding P values were derived from different GWAS models, each being fitted for each trait of interest separately in the respective training set. In order to correct for population stratification and relationships between individuals, a principal component analysis (PCA) was performed on genomic data and significance among principal components (PC) was tested in advance with a Tracy Widom test as implemented in the program EIGENSTRAT [30]. Then, the PC with P values ≤10^−100^ (or ≤0.05) were used as fixed covariates in single-SNP GWAS runs. The resulting genomic relationship matrix was denoted as **G**_**P**100_ (or **G**_**P**005_). Genomic relationship matrices with weighting factors based on results from single-SNP GWAS may not adequately represent or may overweight regions because different SNPs can capture the effect from the same QTL due to long-range LD. However, a SNP effect is not corrected for any other SNP effect in a single-marker regression type GWAS. We also investigated the usefulness of weighting the **G** matrix with results from a random-regression BLUP (RRBLUP) in which random SNP effects are fitted simultaneously. Thus, for matrix **G**_**S**_, we used the squares of the estimated SNP effects of the respective trait as weighting factors to build matrix **D** (as was done in [28]). Finally, we also investigated BLUP|GA [17] in this study. To account for genetic architecture, the trait-specific genomic relationship matrix **G**_**z**_ was constructed as a weighted sum of a genetic architecture matrix **S** and a realized relationship matrix **G**_**I**_ (i.e. **G**_**z**_ = ω**S** + (1 − ω)**G**_**I**_). The construction of the **S** matrix was similar to the construction of **G**_**S**_, but it was based only on selected SNPs according to the size of their absolute SNP effects (top%) from RRBLUP. The optimal choices for top% and *ω* were identified with a grid search strategy applied in the training population. The combinations for searching for optimal parameters were the same as in the original study of Zhang et al. [17] (top% within a range of [0.05, 10] and *ω* within the range [0.1, 0.99]). To make sure that the weighted **G** matrices were in the same scale as **G**_**I**_, all weighting factors were divided by their mean. To mimic the real situation in the best way and avoid over-fitting, all weighting factors in all models were derived exclusively from individuals in the respective training set. To assess whether focusing on functional information improves prediction accuracy, the original GBLUP was applied to the functional subset of the WGS data (HD array data) by building a genomic relationship matrix **G**_**G**_ based on WGS_genic data (HD_genic data) with weights in **D** being 1.

Each approach mentioned above was investigated using fivefold random cross-validation (i.e. having 614 or 615 individuals in the training set and 178 or 179 individuals in the validation set) with five replications and was applied to both WGS and HD array data. Predictive ability was measured as the correlation between the obtained direct genomic values (DGV) and DRP for each trait of interest. DGV and corresponding variance components were estimated using ASReml 3.0 [31].

In layer chicken breeding, genomic breeding values are especially interesting for selecting the best individuals from full-sib families. Thus, we performed the Spearman’s rank correlation to evaluate the ranking of full-sibs according to DRP and DGV in a randomly chosen full-sib family with 12 individuals. Results presented here were from the validation sets of the first replicate of a fivefold cross-validation.

## Results and discussion

### Data summary

**Fig. 1:**
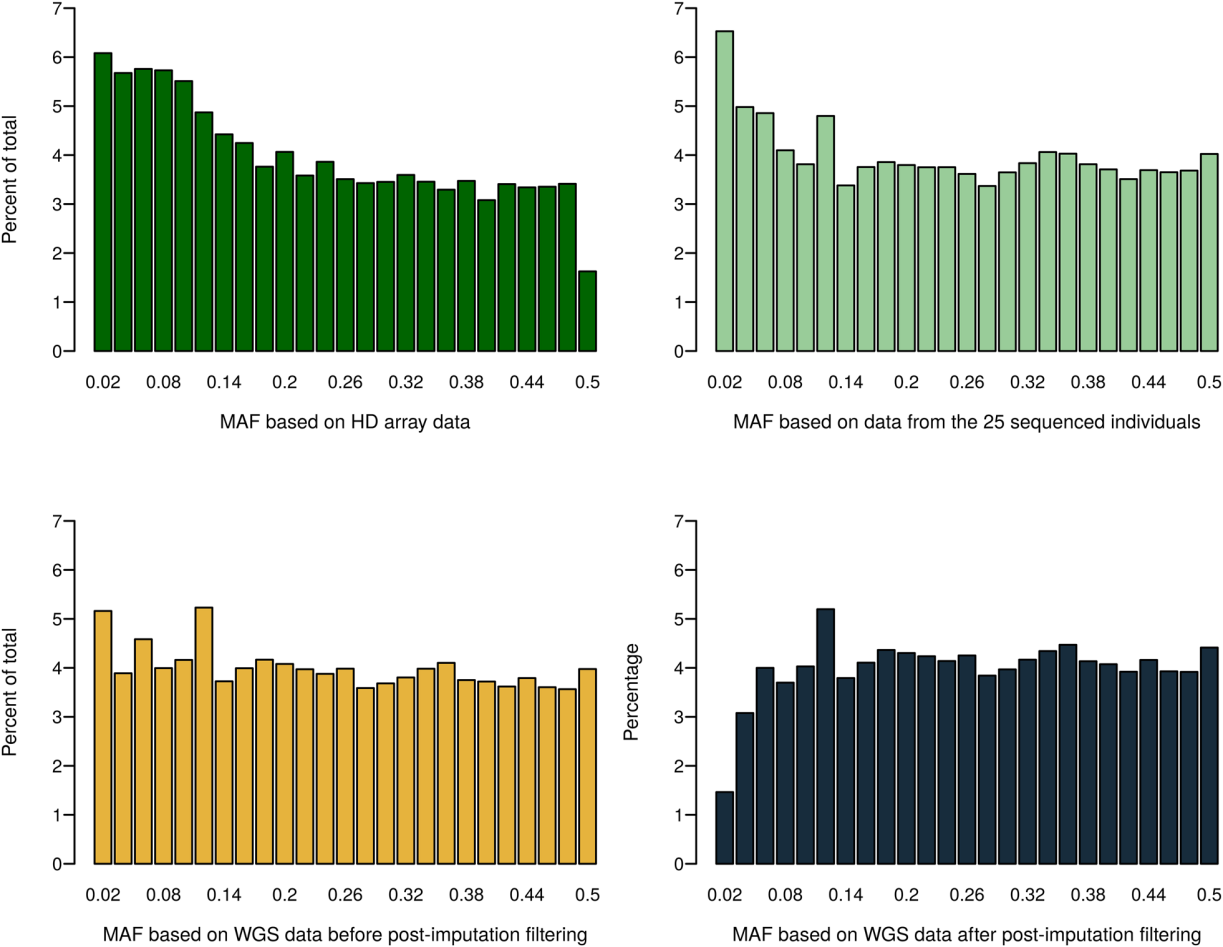
Percentage of SNPs in each MAF bin for high-density (HD) array data and data from re-sequencing runs of the 25 sequenced chickens (*top*), and for imputed whole-genome sequence (WGS) data after imputation and after post-imputation filtering (*bottom*). The values on the x-axis are the upper limit of the respective bin

Numbers of SNPs in different MAF bins for different datasets are shown in Fig. 1. The difference in the distribution of SNPs between HD array data and data from re-sequencing runs is illustrated in the top panel. The last bin (0.48 < MAF ≤ 0.5) contains only half the number of SNPs since, in this bin, only one allele frequency class (25 out of 50 alleles) is represented, while in all other bins two frequency classes (e.g. 24 and 26 out of 50 alleles in the adjacent class) are reflected. The MAF distribution based on WGS data was significantly different from that based on HD data (tested with a *χ* ^2^-test, P < 0.001). For data from re-sequencing runs of the 25 sequenced chickens, the number of SNPs per bin decreased with increasing MAF. SNPs with a very small MAF are not so extremely overrepresented in the re-sequenced set as in other studies with sequenced data [32, 33], which could be due to two reasons. First, the size of the reference dataset was relatively small (25 chickens) and thus, some of the rare variants may not be captured. Second, the commercial layers have been subject to intensive within-line selection, which might have reduced the genetic diversity dramatically, and further resulted in a lack of rare SNPs [34]. Presumably, this problem can only be overcome with a larger sequenced reference set, which would allow higher imputation accuracies for rare SNPs. Numbers of SNPs in different MAF bins in the WGS data set before and after post-imputation filtering are in the bottom panel of Fig. 1. Unlike Van Binsbergen et al. [10], in which 429 sequenced individuals from several cattle breeds were used as a reference set for imputation process, we did not observe a clear U-shaped distribution of MAF in the imputed WGS data. This means that some of the rare SNPs in the re-sequenced individuals were either not present in all the other individuals of the population or got lost during the imputation process, partly because of the poor imputation accuracy for SNPs with a low MAF [35, 36]. Starting from more than 9 million SNPs after imputation (monomorphic SNPs excluded), 200,679 SNPs were filtered out due to a low MAF, and 85% of these filtered SNPs had low imputation accuracy (Rsq of minimac3 <0.8) as well, which means that SNPs with a low MAF are even less represented in the SNP set. Furthermore, 1.3 million SNPs among the imputed SNP set, which passed the MAF criteria, were filtered out due to low imputation accuracy only; these were evenly distributed over all MAF bins. In total, more than 50% of SNPs were filtered out due to low imputation accuracy in the leftmost three MAF bins (0 < MAF ≤ 0.06). The fact that we found high rates of low Rsq values within the set of SNPs with a low MAF could be due to low LD between these SNPs and adjacent SNPs, which can result in lower imputation accuracy [for imputation accuracies in different MAF bins (see Additional file 2: Figure S1)] [37–41]. Filtering out a large number of SNPs with a low MAF—in many cases, because imputation accuracy is too low—could weaken the advantage of imputed WGS data, which contain a large number of rare SNPs [6], although GP with all imputed SNPs without quality-based filtering did not improve the prediction ability in our case (results not shown). In addition, LD pruning was not performed in our study, because in a preliminary study we found that predictive ability based on the pruned dataset was the same as that based on data without pruning (results not shown).

### Comparison between HD array data and WGS data using different weighting factors

**Fig. 2:**
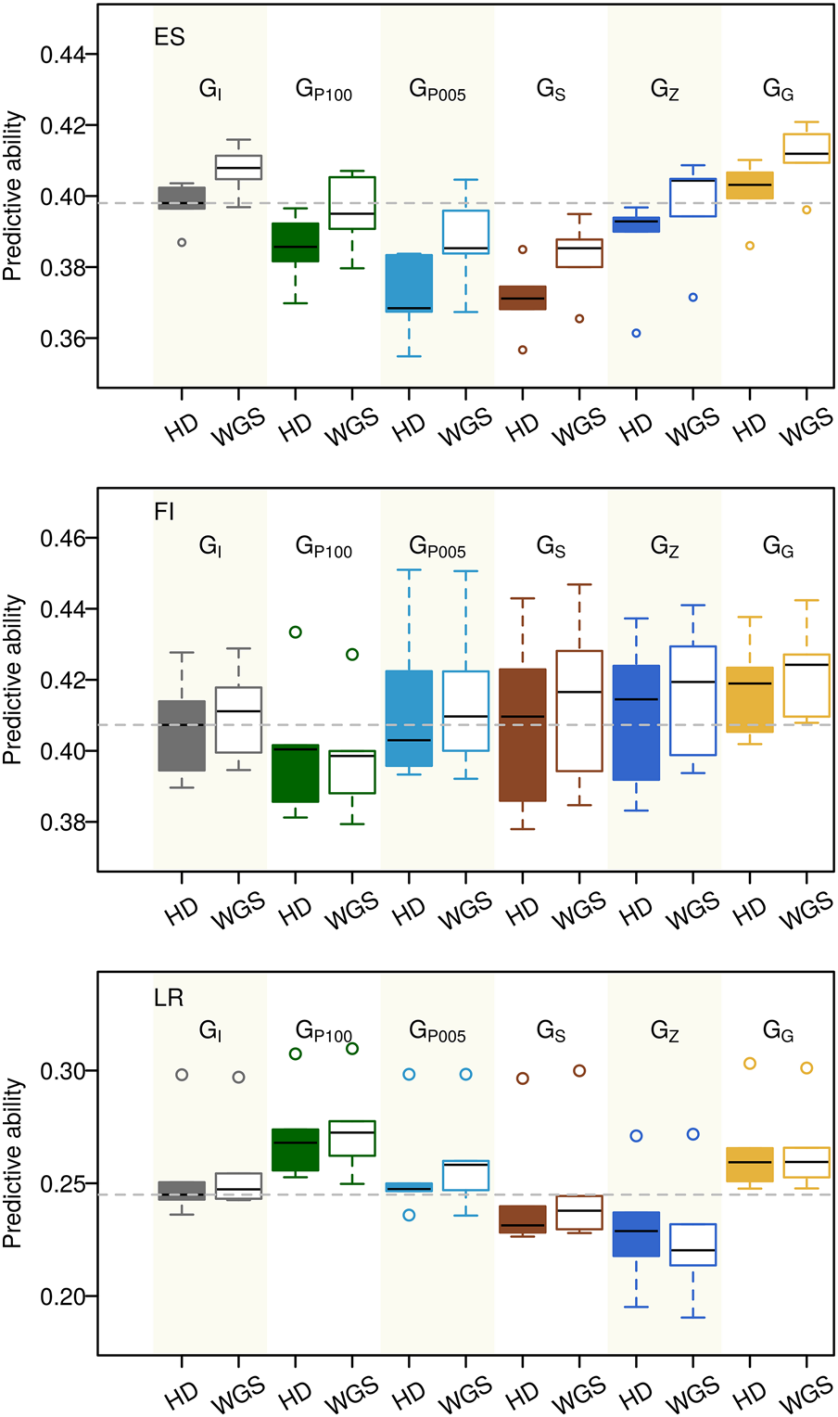
Predictive ability obtained with GBLUP using different weighting factors based on high-density array data and whole-genome sequencing data. The predictive ability was measured as the correlation between direct genomic breeding values (DGV) and de-regressed proofs (DRP) in the validation set. Results are for the traits eggshell strength (ES), feed intake (FI) and arcsine transformed laying rate in the last third of the laying period (LR). HD stands for high-density data and WGS stands for whole-genome sequencing data. **G**_**I**_ stands for the original GBLUP, **G**_**P**_ represents the model with −(log_10_ *P*) from GWAS as weighting factors where principal components that were significant at P values ≤ 10^−100^ (≤ 0.05) were used as covariates in the GWAS model, denoted as **P**_100_ (**P**_005_). **G**_**S**_ stands for the GBLUP model with the squares of estimated SNPs effect as weighing factors. **G**_**z**_ stands for the results from BLUP|GA as described in the “Methods” section. **G**_**G**_ stands for the original GBLUP but only based on genic SNPs. The *dashed horizontal line* denotes the median predictive ability of GBLUP with HD data as a reference. *Note* that all the outliers for trait LR were from the same replicate

Predictive abilities obtained with GBLUP using different weighting factors based on HD array data and WGS data are in Fig. 2 for the traits ES, FI, and LR, respectively. Predictive ability was defined as the correlation between DGV and DRP of individuals in the validation set. Generally speaking, predictive ability could not be clearly increased when using WGS data compared to HD array data regardless of the different weighting factors studied. Using genic SNPs from WGS data had a positive effect on prediction ability in our study design.

Averaging over the three traits analyzed here, the predictive ability ± standard deviation for the original GBLUP was 0.353 ± 0.074 based on HD array data and 0.358 ± 0.076 based on WGS data. When −(log_10_ *P*) (with P values from GWAS with different covariates in the model) were used as weighting factors, predictive abilities for G_P100_ (G_005_) were 0.352 ± 0.062 (0.347 ± 0.072) based on HD array data and 0.356 ± 0.062 (0.354 ± 0.073) based on WGS data. Unlike the SNP effects that were estimated from RRBLUP, in which effects are assessed simultaneously, SNP effects were estimated independently in GWAS. Thus, effects of a group of SNPs which represent the same QTL could not be fitted simultaneously, and thus the overall weighting of a region might depend on the marker density. De los Campos et al. [15] studied a public human type-2 diabetes case–control dataset that included genotype data from a 500 K SNP platform and around 6000 phenotype records from unrelated individuals. They reported that the predictive reliability (square of predictive accuracy) with a prediction model weighted by −(log_10_ *P*) increased by a factor of 110% compared to that with the original GBLUP. Similarly, Su et al. [28] reported that predictive ability using −(log_10_ *P*) as weighting factors was higher than that obtained with the original GBLUP, based on more than 5000 Nordic Holstein bulls that were genotyped with the Illumina Bovine SNP50 BeadChip. However, the improvement in predictive ability by using −(log_10_ *P*) as weighting factors in GP was not observed in our dataset.

**Fig. 3:**
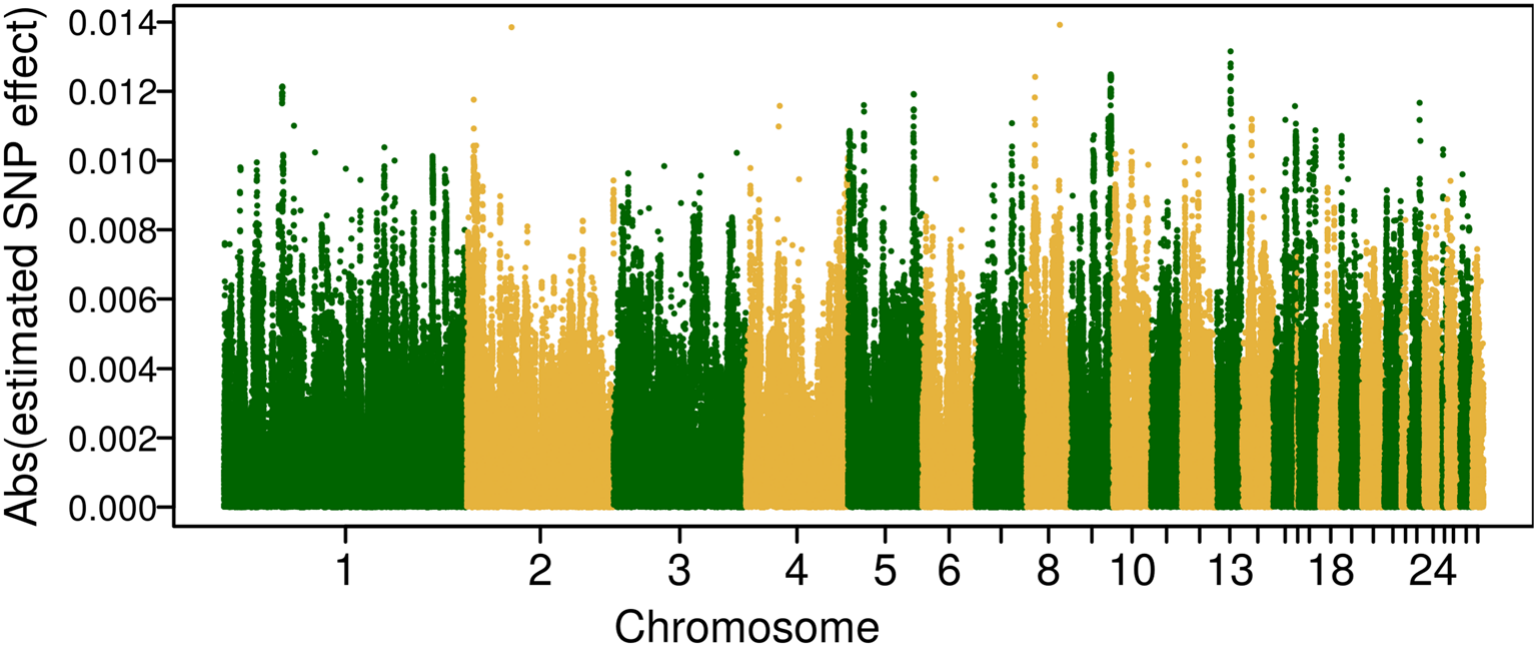
Manhattan plot of absolute estimated SNP effects for trait eggshell strength based on high-density (HD) array data. SNP effects were obtained from RRBLUP in the training set of the first replicate

**Fig. 4:**
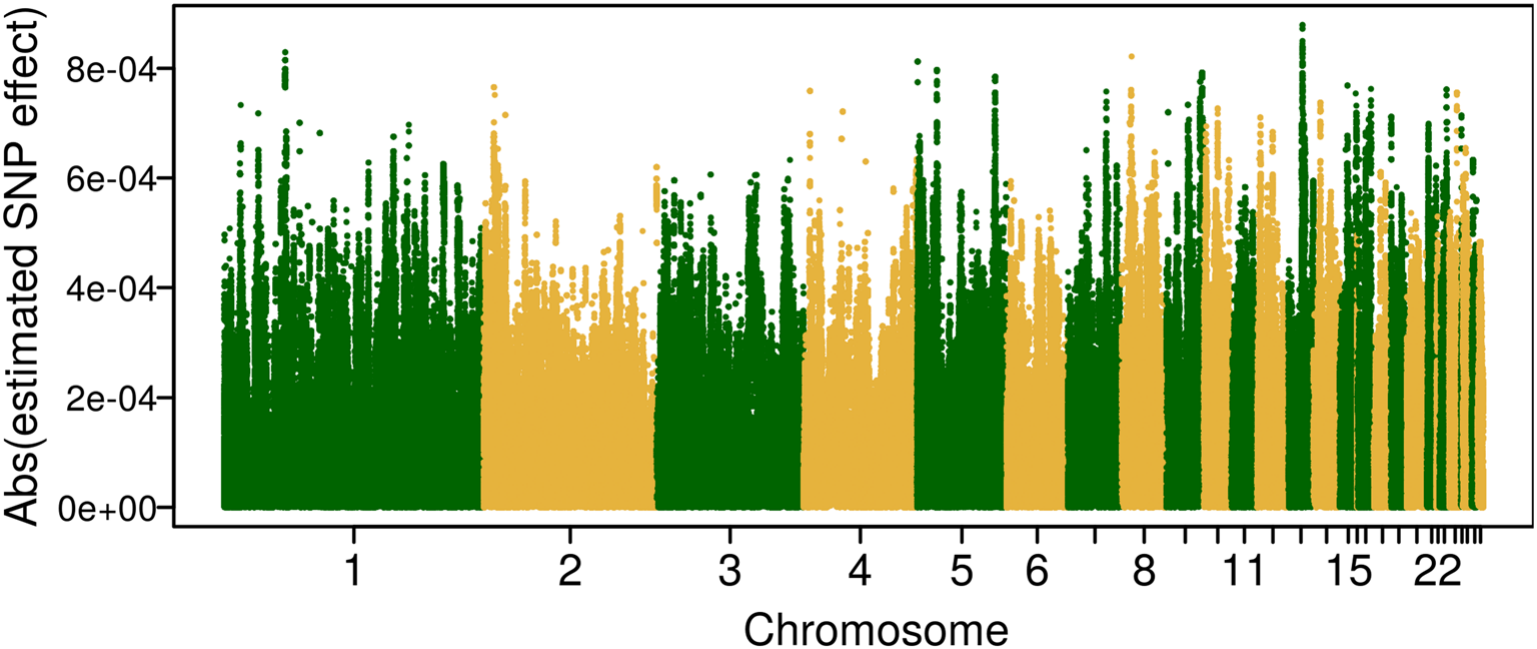
Manhattan plot of absolute estimated SNP effects for trait eggshell strength based on whole-genome sequence (WGS) data. SNP effects were obtained from RRBLUP in the training set of the first replicate

Furthermore, using the squares of SNP effects as weighting factors in GBLUP (**G**_**S**_) resulted in slightly lower predictive abilities compared to the original GBLUP, in both analyses based on HD array data and on WGS data, respectively, as shown in Fig. 2. For **G**_**S**_, averaging over the three traits, predictive ability was 0.341 ± 0.076 based on HD data and 0.348 ± 0.078 based on WGS array data, compared to 0.353 ± 0.074 (for HD array data) and 0.358 ± 0.076 (for WGS data) with the original GBLUP. These results are in agreement with Su et al. [28], who reported that GBLUP with the squares of SNP effects as weighting factors did not improve predictive ability compared to the original GBLUP or to the model with −(log_10_ *P*) as weighting factors. The lack of improvement in predictive ability when using the squares of SNP effects as weighting factors might be due to two reasons. One reason is the occurrence of sequencing or imputation errors, i.e. in our study, the most probable genotypes imputed from Minimac3 were used as WGS data rather than genotype probabilities, which does not account for the uncertainty of imputation. The second reason is that the noise and uncertainty of estimated SNP effects could also bias predictive ability [28]. In our study, DGV of the training population were assigned to millions of SNPs [Figs. 3, 4; (Additional file 3: Figure S2)], so that the effect of each SNP was very small. However, the prediction error of a SNP effect might be even larger than the SNP effect itself. In addition, the size of the training set was relatively small, which could further enhance the uncertainty of SNP effects. Thus, the combination of both mentioned reasons could lead to lower predictive ability, since the DGV of individual *i* is the summation of estimated SNP effects times its genotypes (i.e. $DGV_{i} = \sum\nolimits_{k = 1}^{m} {X_{ik} \beta_{k} }$).

With BLUP|GA, predictive ability was 0.342 (±0.085) based on HD array data and 0.346 (±0.091) based on WGS data averaged over the three traits analyzed (Fig. 2). Generally speaking, BLUP|GA did not improve predictive ability with WGS or HD data, compared to the original GBLUP. Zhang et al. [17] reported that BLUP|GA outperformed the original GBLUP for production traits (i.e. fat percentage and milk yield) in a German Holstein cattle population, while its performance was similar to that of GBLUP for SCS. A well-known candidate gene *DGAT1* has a strong influence on fat percentage [42, 43], while for SCS no major genes are known. This suggests that BLUP|GA is especially useful when QTL regions that heavily influence the trait are present in the genome. The genetic architecture of ES, FI, and LR seems to be more similar to that of SCS than of fat percentage which might explain why no strong candidate genes have been identified to date and also that no strong SNP effects have been detected in the GWAS runs performed in this study (see Additional file 4: Figure S3). The SNP effects estimated from RRBLUP based on HD array (WGS) data are in Fig. 3 (Fig. 4) and further illustrate that ES, FI, and LR are controlled by numerous SNPs with very small effects.

**Fig. 5:**
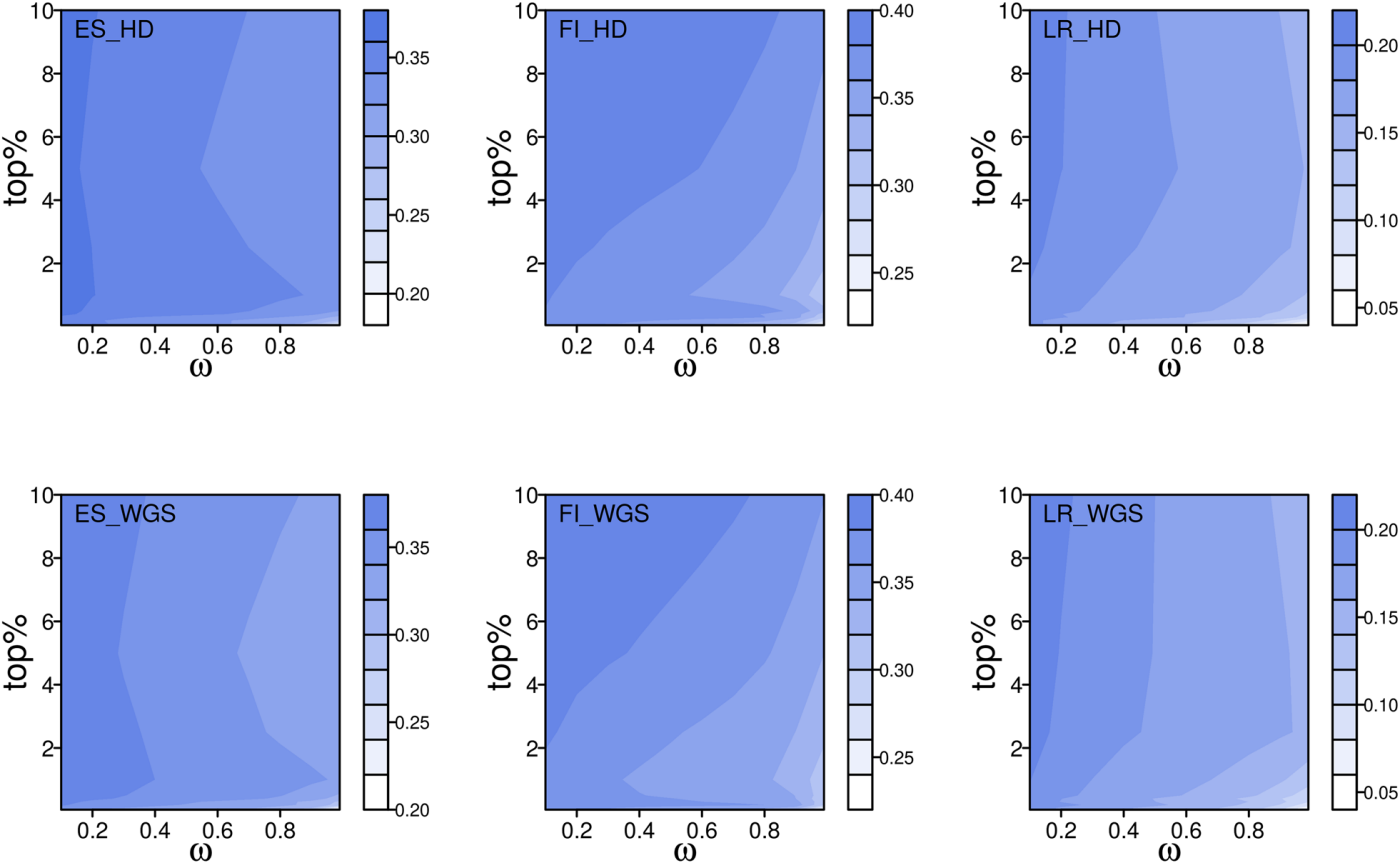
Predictive ability of the best linear unbiased prediction given the genetic architecture (BLUP|GA) in the training stage to select the optimal parameter combination for the application stage. Predictive ability in this figure is the mean correlation between direct genomic breeding values (DGV) and de-regressed proofs (DRP). The first row is for high-density (HD) array data, while the second row is for whole-genome sequence (WGS) data. The x-axis stands for the overall weighting factor; y-axis stands for the percentage of SNPs selected based on the SNP effects (top%); different colors stand for different levels of predictive ability

When focusing on the training stage of BLUP|GA, the burden of calculation to identify the optimal combination for parameters top% and *ω* with a grid strategy was huge. Prediction abilities of BLUP|GA in the training stage are in Fig. 5 for each parameter combination exemplarily for the first fold of the first replicate. The combination of large *ω* and small top% tended to give lower predictive ability. As top% increased and *ω* decreased, predictive ability tended to increase. In most cases, the optimal option for *ω* based on HD data and WGS data was 0.1 in our study, which is the minimal *ω* we analyzed. The optimal option for top% was 10%, which is the maximal top% we analyzed, and is different from the findings of Zhang et al. [17]. These authors tended to select a smaller top% while there was no obvious pattern in the selection of *ω*. Those 10% SNPs explained approximately 23% of the total variance of SNP effects for ES. Optimal combinations in each fivefold cross-validation of each replicate for each trait are in Additional file 5: Table S2 and Additional file 6: Table S3. It should be noted that, as described in Zhang et al. [17], accuracy of GP based on the optimal parameters obtained in the training stage by cross-validation may not lead to the highest accuracy in the application stage.

Averaging over the three traits analyzed here, predictive ability ± standard deviation was 0.366 ± 0.075 based on the WGS_genic data and 0.361 ± 0.072 based on HD_genic data, compared to 0.353 (HD array data) and 0.358 (WGS data), which means that GP with WGS_genic resulted in the highest predictive ability in our study. Similarly, Do et al. [44] reported that predictive ability increased only when SNPs in genes were considered for residual feed intake based on 1272 Duroc pigs, which were genotyped with the 60 K SNP chip, although the increase was not significantly different from that obtained with 1000 randomly SNPs. In chicken, Morota et al. [12] studied predictive ability with 1351 commercial broiler chickens genotyped with the Affymetrix 600 K chip, and found that prediction based on SNPs in or around genes did not result in a higher accuracy using kernel-based Bayesian ridge regression. In our dataset, predictive ability with HD_genic data was slightly higher than that with all HD data. Furthermore, the benefit was observed when using WGS_genic, which could be due to the fact that using only genic SNPs reduces the noise in WGS data and might increase the chance to identify the potential causal mutations. Koufariotis et al. [45] found that significant SNPs in the GWAS were enriched in coding regions based on 17,425 Holstein or Jersey bulls and cows, which were genotyped with the 777 K Illumina Bovine HD array. The enrichment of significant SNPs could further imply that using genic SNPs can help us to achieve higher predictive ability.

The bias of DGV was assessed as the slope coefficient of the linear regressions of DRP on DGV within the validation sets of random fivefold cross-validation. The averaged regression coefficient ranged from 0.520 (G_P005_ of HD dataset) to 0.871 (G_I_ of WGS dataset) for the trait ES (see Additional file 7: Figure S4). No major differences were observed between using HD and WGS datasets within different methods. Generally, regression coefficients were all smaller than 1, which means that the variance of the breeding values tends to be overestimated. However, the regression coefficients were closer to 1 when the identity matrix was used in the prediction model (i.e.** G**_**I**_, **G**_**G**_). The overestimation could be due to the fact that those analyses were based on cross-validation where the relationship between training and validation populations might cause a bias. Another possible reason for the overestimation could be that, in this chicken population, individuals were under strong within-line selection. The same tendency was observed for traits FI and LR (results not shown).

### Comparison within a full-sib family

**Fig. 6:**
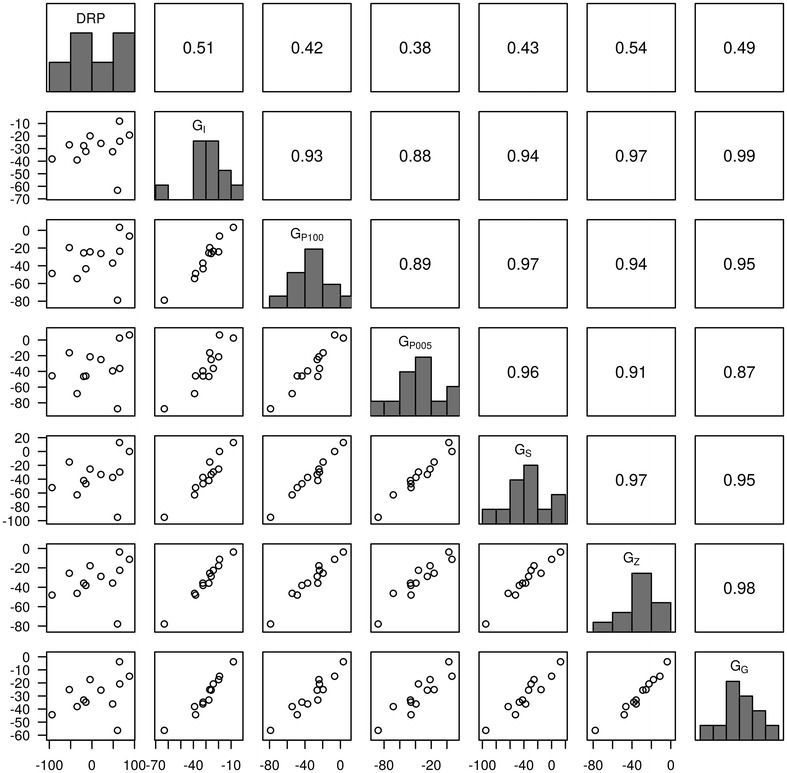
Predictive ability in a full-sib family with 12 individuals for eggshell strength based on high-density (HD) array data of one replicate. In each plot matrix, the diagonal shows the histograms of DRP and DGV obtained with various matrices. The *upper triangle* shows the Spearman’s rank correlation between DGV with different matrices and with DRP. The *lower triangle* shows the scatter plot of DGV with different matrices and DRP

 To get an insight into the ranking of 12 full-sibs within a family according to DRP and DGV, DGV that were predicted in the validation sets with different **G** matrices in the first of the five replicates of the cross-validation runs are in Figs. 6 (HD data) and 7 (WGS data) for ES, and Additional file 8: Figure S5 and Additional file 9: Figure S6 for traits FI and LR, respectively. The higher the rank correlation is, the higher is the possibility to select the same candidates. Based on HD array data, DGV from different weighting models had a relatively high rank correlation with those from **G**_**I**_ (from 0.88 to 0.97 for ES). This suggested that the same candidate tended to be selected in different models. Likewise, the rank correlations based on WGS data were relatively high as well, with minimal values of 0.91 between **G**_**G**_ and **G**_**P**005_. In addition, the Spearman’s rank correlation between **G**_**I**_ based on HD array data and that based on WGS data was 0.98. Spearman’s rank correlation between **G**_**G**_ with WGS_genic data and **G**_**I**_ with WGS data was 0.99, which indicated that there was hardly any difference in selecting candidates based on HD array data, or WGS data, or WGS_genic data with GBLUP. Generally, the same set of candidates tended to be selected regardless of the dataset (HD array data or WGS data) and weighting factors (identity weights, squares of SNPs effect, or P values from GWAS) used in the model. When comparing the DGV from different models with DRP, the Spearman’s rank correlations were modest (from 0.38 to 0.54 with HD data and from 0.31 to 0.50 with WGS data) and within the expected range considering the overall predictive ability obtained in the cross-validation study (see Fig. 2). Although DGV from different models were highly correlated, Spearman’s rank correlation of the respective DGV to DRP clearly varied. This fact, however, should not be overvalued regarding the small sample size that was used here (n = 12) and the fact that the DGV of the full-sib family were estimated from different CV folds. Thus, a forward prediction was performed with 146 individuals from the last two generations as validation set. In this case the same tendency was observed, namely that DGV from different models were highly correlated within a large half-sib family. However, in this forward prediction scenario, the predictive ability with genic SNPs was slightly lower than that with all SNPs (results not shown).

**Fig. 7:**
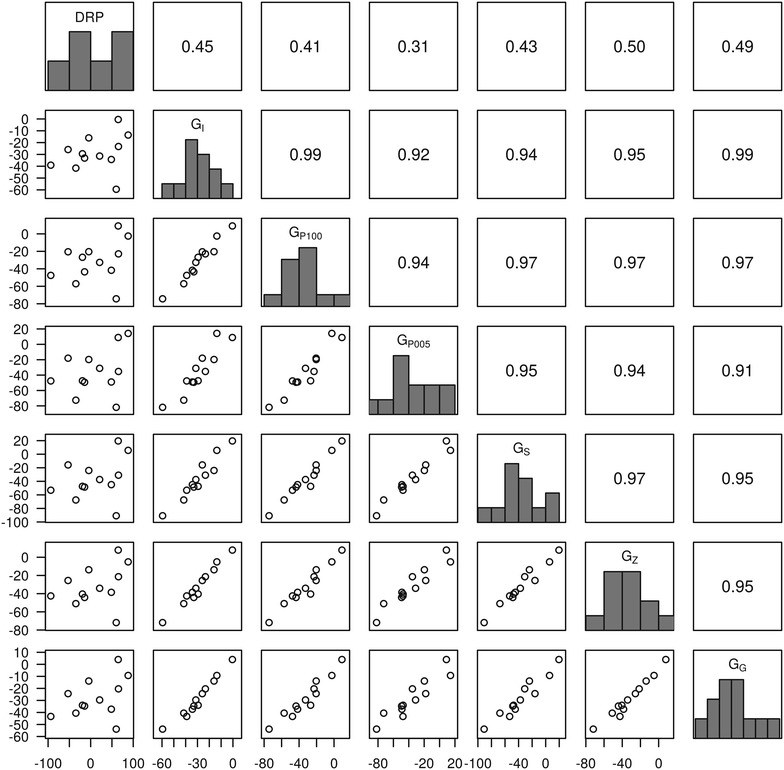
Predictive ability in a full-sib family with 12 individuals for eggshell strength based on whole-genome sequence (WGS) data of one replicate. In each plot matrix, the diagonal shows the histograms of DRP and DGV obtained with various matrices. The *upper triangle* shows the Spearman’s rank correlation between DGV with different matrices and with DRP. The *lower triangle* shows the scatter plot of DGV with different matrices and DRP

### Perspectives and implications

Using WGS data in GP was expected to lead to higher predictive ability, since WGS data should include most of the causal mutations that influence the trait and prediction is much less limited by LD between SNPs and causal mutations. Contrary to this expectation, little gain was found in our study. One possible reason could be that QTL effects were not estimated properly, due to the relatively small dataset (892 chickens) with imputed WGS data [18]. Imputation has been widely used in many livestock [38, 46–48], however, the magnitude of the potential imputation errors remains difficult to detect. In fact, Van Binsbergen et al. [10] reported from a study based on data of more than 5000 Holstein–Friesian bulls that predictive ability was lower with imputed HD array data than with the actual genotyped HD array data, which confirms our assumption that imputation could lead to lower predictive ability. In addition, discrete genotype data were used as imputed WGS data in this study, instead of genotype probabilities which can account for the uncertainty of imputation and may be more informative [49]. At present, sequencing all individuals in a population is not realistic. In practice, there is a trade-off between predictive ability and cost efficiency. When focusing on the post-imputation filtering criteria, the threshold for imputation accuracy was 0.8 in our study to guarantee the high quality of the imputed WGS data. Numerous rare SNPs, however, were filtered out due to the low imputation accuracy as shown in Fig. 1 and Additional file 2: Figure S1. This could increase the risk of excluding rare causal mutations. However, Ober et al. [8] did not observe an increase in predictive ability for starvation resistance when rare SNPs were included in the GBLUP based on ~2.5 million SNPs that had been identified from 192 *D. melanogaster*. Further investigation needs to be done in chicken, especially when more founder sequences become available.

Another reason why we did not observe any increase in predictive ability when using WGS data could be that we did not apply variable selection. The density of WGS data was around 15 times higher than that of HD array data, which increased LD between SNPs. Thus, QTL effects were assigned to more SNPs in WGS data than in HD array data, which could be overcome by variable selection. Su et al. [28] reported that reliability of GP increased by more than 5% when grouping 30 adjacent SNPs. In each group, a common weight was assigned which reflected the mean over the SNP effects in the same group. In addition, Brøndum et al. [11] reported that the reliability of GP can be improved by adding several significant QTL into the regular bovine 54 K array data. In our study, 20 top SNPs were selected according to their estimated effects from RRBLUP or −(log_10_ *P*) of GWAS and used as fixed effects in GBLUP, but it did not improve predictive ability (results not shown). GP with genic SNPs from WGS (the WGS_genic data) provided the highest predictive ability compared to that obtained when all SNPs from WGS data were used. This implies that selecting the proper variables could help us to reduce noise and increase predictive ability. Using a variable selection model may also help. Based on a simulated WGS data, Wimmer et al. [50] reported that feature selection methods (e.g. the least absolute shrinkage and selection operator) have limitations when the ratio between sample size and number of SNPs is unfavorable, which was the case in our study. A similar conclusion was drawn by Heidaritabar et al. [14] who found that there was no advantage in genomic prediction with a BayesC model compared to GBLUP based on imputed WGS data of 1244 white layer chickens. In addition, Ober et al. [8] observed no differences in predictive ability with BayesC and GBLUP based on a dataset with ~2.5 million SNPs that were identified for a *D. melanogaster* population. In addition, with the increasing knowledge about gene networks, pathways and suitable prediction models, blending biological knowledge based on gene annotations and complex interactions may provide insights to guide GP [51].

Our fourth possible explanation for the small improvement in predictive ability with WGS data refers to the population structure. Commercial chickens have been subject to intensive within-line selection, which has a strong effect on the population structure. MacLeod et al. [52] studied the accuracy of GP based on WGS data for two simulated populations with a different demographic history. They found that in a highly selected population with a small effective population size there was almost no gain in prediction accuracy when using WGS data compared to HD data, which is in agreement with our findings. The way the data were split for the cross-validation strategy might enhance this effect compared to a forward prediction scenario.

The use of incomplete WGS information could also weaken its predictive ability. First, in most studies, sex chromosomes were disregarded in the GP scheme, considering that the transmission of sex chromosomes and that of autosomes differ and that the density of SNPs and LD structure on the sex chromosomes is lower than on autosomes in commercial SNP chips. However, recent studies have discovered an increasing number of genes on the sex chromosomes that affect economic traits. For example, Su et al. [53] found that including the sex chromosomes in the GP scheme could increase the predictive ability averaged over 15 traits that were included in the Nordic Total Merit index (e.g. milk yield and fat yield). Second, WGS data, technically include all DNA variants [e.g. copy number variations (CNV) and InDels], but the studies on GP in livestock have so far mostly focused on SNPs. However, according to previous studies [54, 55], CNV and other types of structural variations play an important role in gene expression and phenotypic variation. Third, although the chicken karyotype consists of 39 chromosomes, data from re-sequencing represent only 30 chromosomes and two linkage groups since the reference genome was not available for some of the micro-chromosomes which are also assumed to be gene-rich [56, 57]. Beyond that, chromosome 16, which hosts the chicken major histocompatibility complex, is included in the reference sequence but has a low marker density [58] and the quality of the reference sequence is expected to be inferior due to the high genetic variability. Furthermore, non-nuclear DNA present in the mitochondria is not accounted for. In general, further work is necessary to assess the importance of the entire DNA variation on the predictive ability of GP in chicken.

## Conclusions

In this study, we compared the ability of genomic prediction using both high-density array data and imputed whole-genome sequencing data. More comparisons were performed based on GBLUP with different genomic relationship matrices to account for the genetic architecture of the three traits analyzed: eggshell strength, feed intake, and laying rate. Our results show that little or no benefit was gained when using all imputed WGS data compared to HD array data with different weighting approaches in the GBLUP model. However, our results suggest that using genic SNPs for genomic prediction has the potential to improve the predictive ability both with HD and WGS data. Overall, the same candidates tend to be selected from a full-sib family of interest regardless of the genotype data and weighting factors used.

## Additional files

**Additional file 1**: **Table S1.** Number of individuals in each generation.

**Additional file 2**: **Figure S1.** Imputation accuracy (Rsq of Minimac3) in each minor allele frequency (MAF) interval.

**Additional file 3**: **Figure S2.** Manhattan plot of absolute estimated SNP effects for traits FI and LR based on high-density (HD) array data and whole-genome sequence (WGS) data, respectively.

**Additional file 4**: **Figure S3.** Manhattan plots of −(log_10_ *P*) for the three traits based on high density array data (panels 1–3) and the whole-genome sequence (WGS) data (panels 4–6). Significance among principal components (PC) was tested in advance with a Tracy Widom test and PC with P values less than 0.05 were used as fixed covariates in single-SNP GWAS runs.

**Additional file 5**: **Table S2.** The optimal parameter in the training stage of BLUP|GA based on HD array data for each fold of fivefold cross-validation in each replicate.

**Additional file 6**: **Table S3.** The optimal parameter in the training stage of BLUP|GA based on WGS data for each fold of fivefold cross-validation in each replicate.

**Additional file 7**: **Figure S4.** Regression coefficient of DGV on genomic prediction using different weighting factors based on high-density array data and whole-genome sequencing data.

**Additional file 8**: **Figure S5.** Predictive ability in a full-sib family with 12 individuals for feed intake based on high-density (HD) array data (top) and whole-genome sequence (WGS) data (bottom) of one replicate. In each plot matrix, the diagonal shows the histograms of DRP and DGV obtained with various **G** matrices. The upper triangle shows the Spearman’s rank correlation between DGV with different **G** matrices and DRP. The lower triangle shows the scatter plot of DGV with different **G** matrices and DRP.

**Additional file 9**: **Figure S6.** Predictive ability in a full-sib family with 12 individuals for laying rate based on high-density (HD) array data (top) and whole-genome sequence (WGS) data (bottom) of one replicate. In each plot matrix, the diagonal shows the histograms of DRP and DGV obtained with various **G** matrices. The upper triangle shows the Spearman’s rank correlation between DGV with different **G** matrices and DRP. The lower triangle shows the scatter plot of DGV with different **G** matrices and DRP.
