# Supplementary material for: Whole-genome sequence-based genomic prediction in laying chickens with different genomic relationship matrices to account for genetic architecture
Source: Genet Sel Evol. 2017 Jan 16;49:8. doi: 10.1186/s12711-016-0277-y (PMC5238523; doi:10.1186/s12711-016-0277-y)
Supplement: Supplementary file 1 — Additional file 1: Table S1. Number of individuals in each generation. [file 12711_2016_277_MOESM1_ESM.docx]

| Generation | 1 | 2 | 3 | 4 | 5 | 6 | Total |
| --- | --- | --- | --- | --- | --- | --- | --- |
| # | 25 | 49 | 49 | 596 | 78 | 78 | 892 |
