# Supplementary figures and images for: Whole-genome sequence-based genomic prediction in laying chickens with different genomic relationship matrices to account for genetic architecture
Source: Genet Sel Evol. 2017 Jan 16;49:8. doi: 10.1186/s12711-016-0277-y (PMC5238523; doi:10.1186/s12711-016-0277-y)

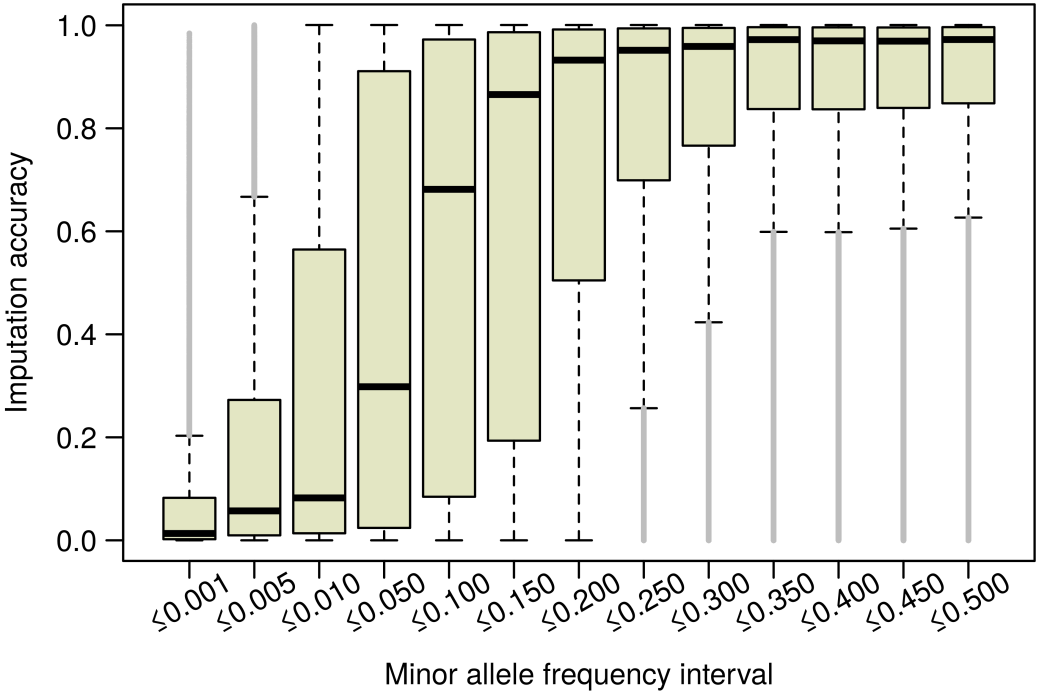

Supplement: Supplementary file 2 — Additional file 2: Figure S1. Imputation accuracy (Rsq of Minimac3) in each minor allele frequency (MAF) interval. [file 12711_2016_277_MOESM2_ESM.docx]

| 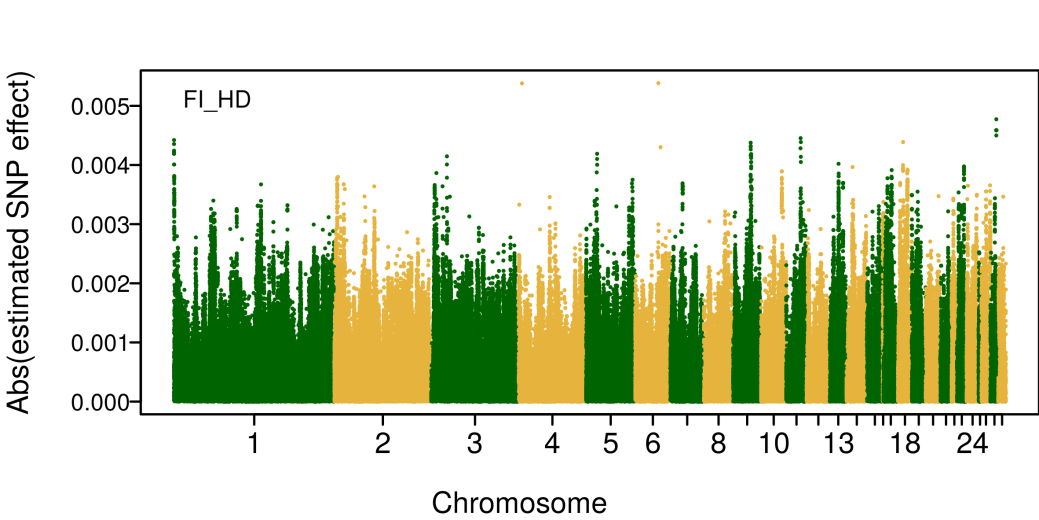 |
| --- |
| 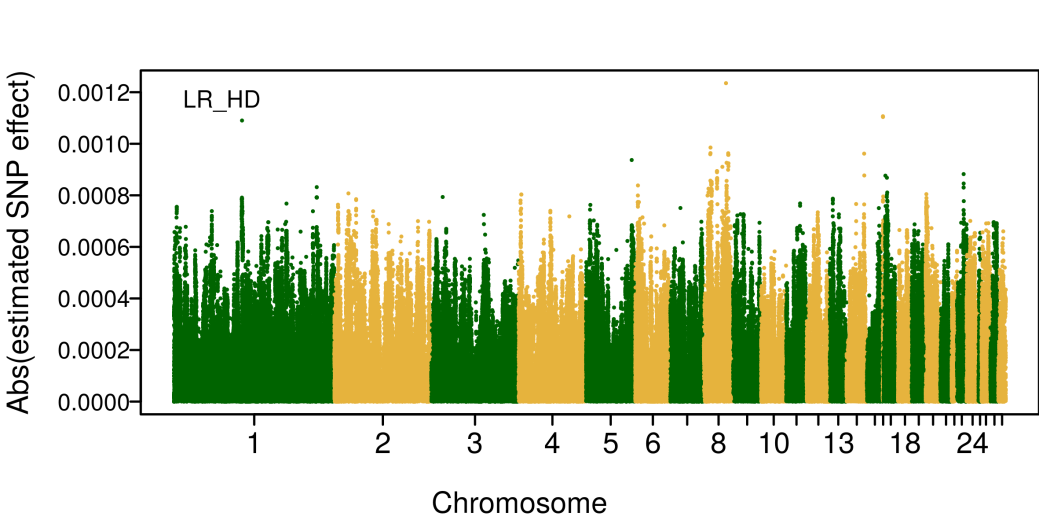 |
| 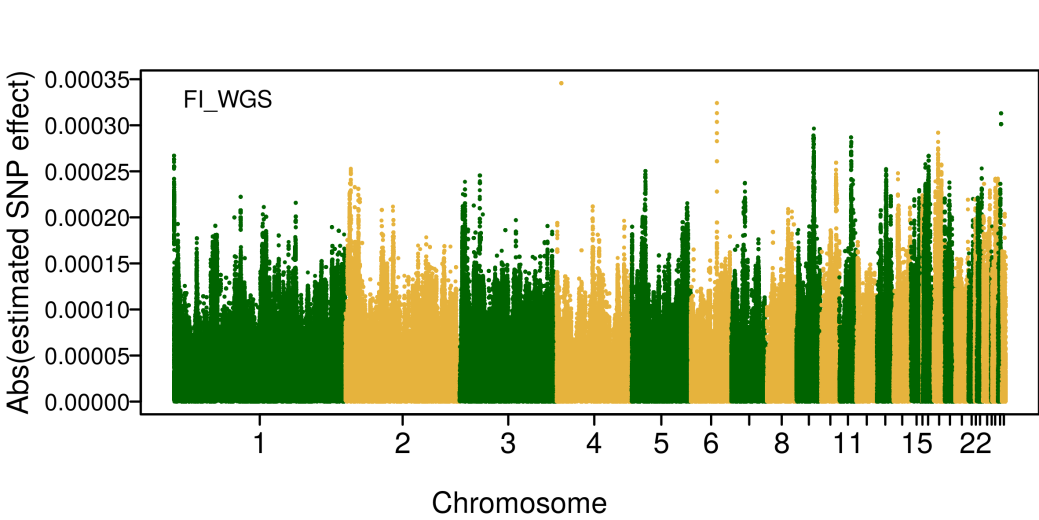 |
| 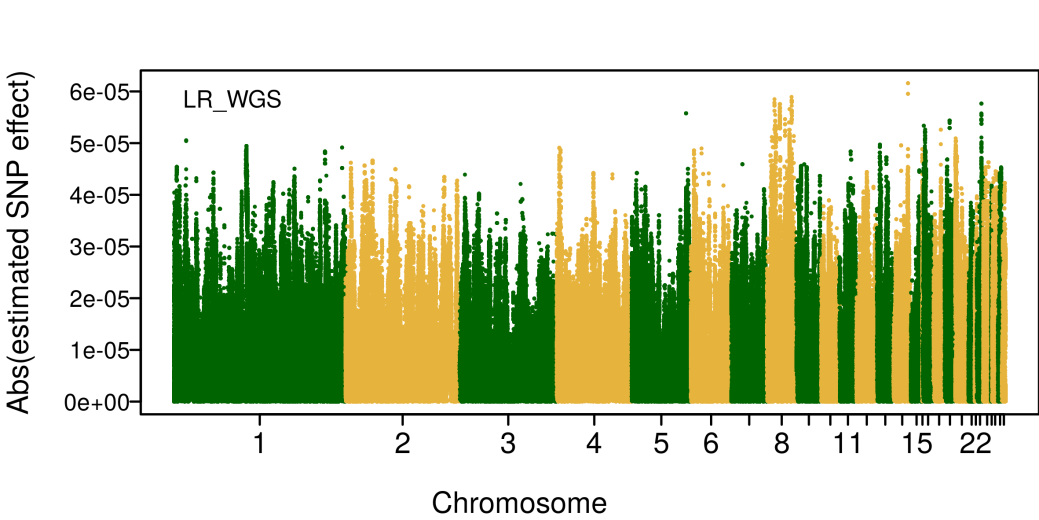 |

Supplement: Supplementary file 3 — Additional file 3: Figure S2. Manhattan plot of absolute estimated SNP effects for traits FI and LR based on high-density (HD) array data and whole-genome sequence (WGS) data, respectively. [file 12711_2016_277_MOESM3_ESM.docx]

| 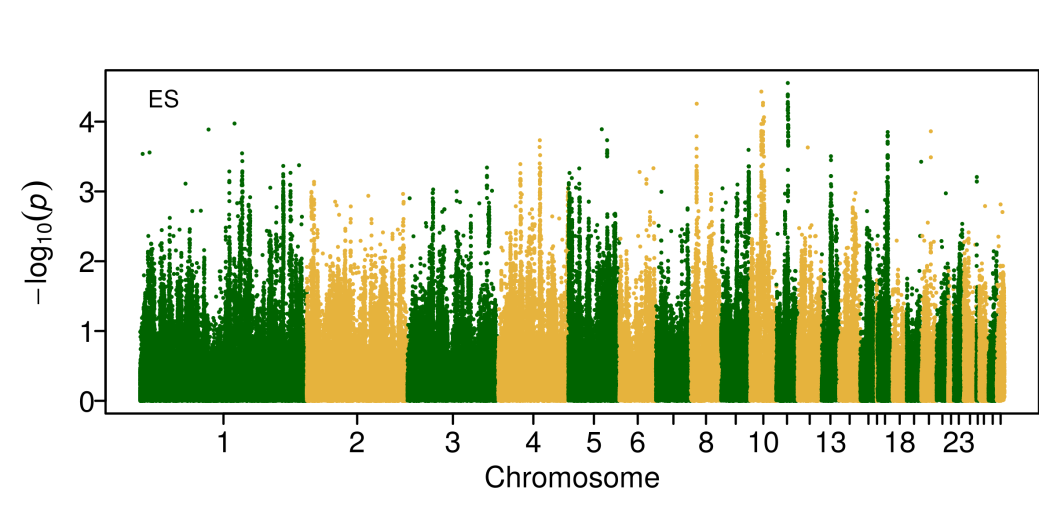 |
| --- |
| 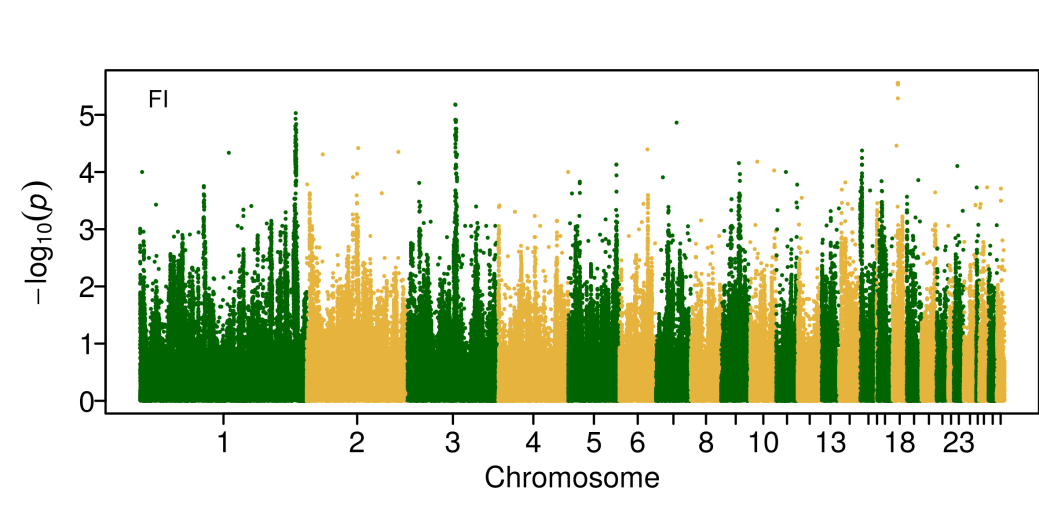 |
| 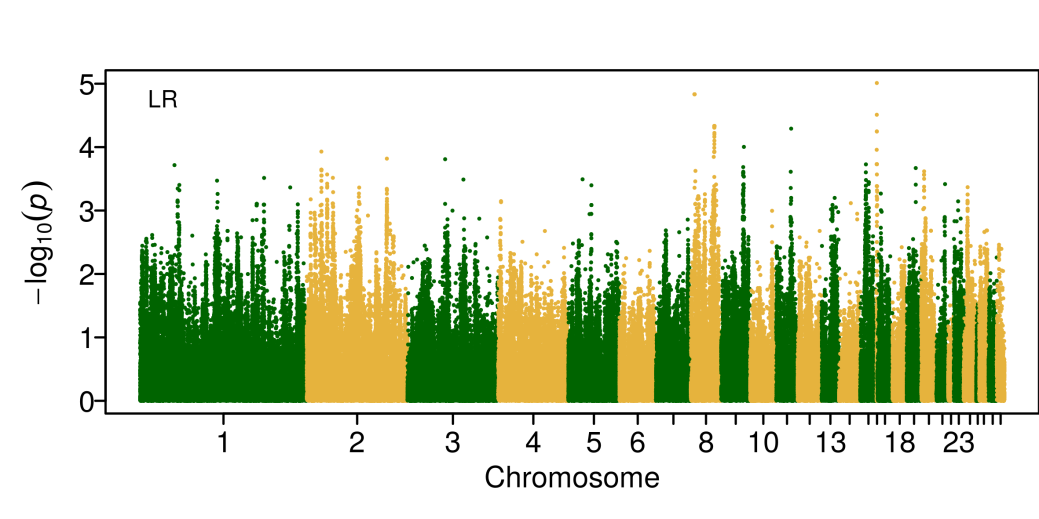 |
| 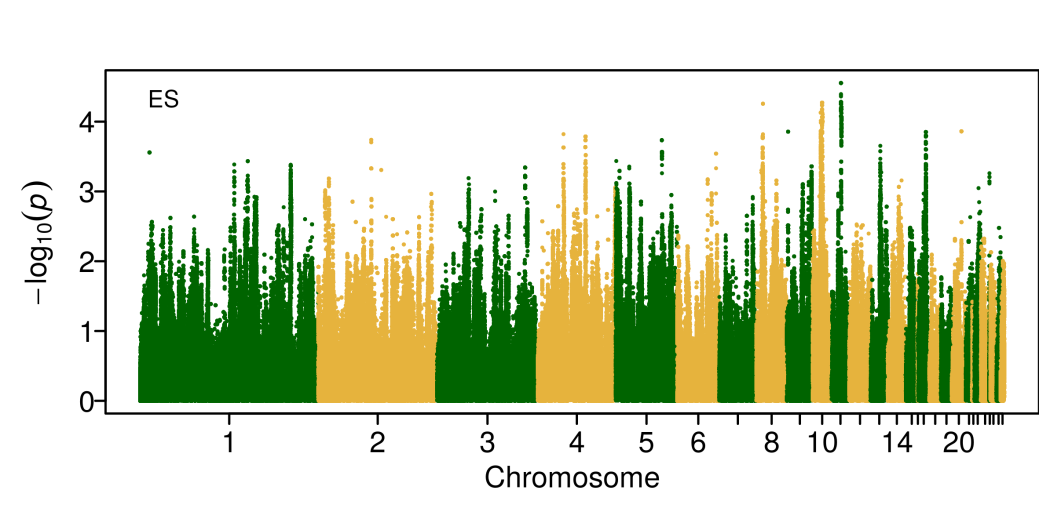 |
| 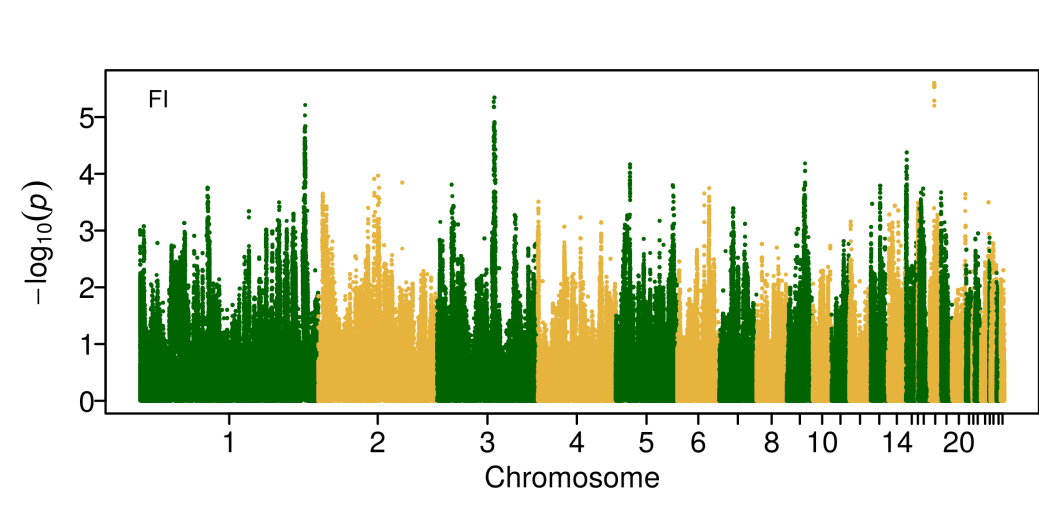 |
| 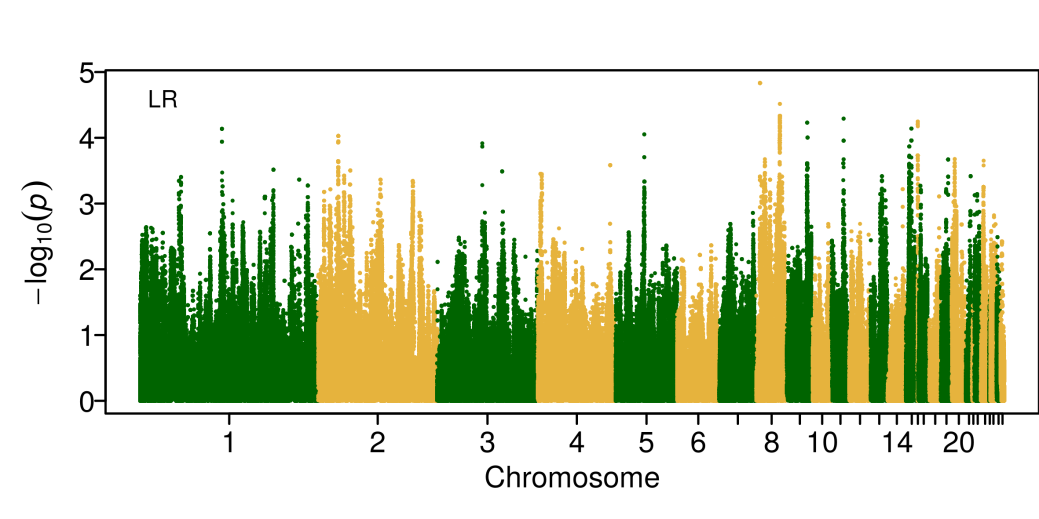 |

Supplement: Supplementary file 4 — Additional file 4: Figure S3. Manhattan plots of −(log10 P) for the three traits based on high density array data (panels 1–3) and the whole-genome sequence (WGS) data (panels 4–6). Significance among principal components (PC) was tested in advance with a Tracy Widom test and PC with P values less than 0.05 were used as fixed covariates in single-SNP GWAS runs. [file 12711_2016_277_MOESM4_ESM.docx]

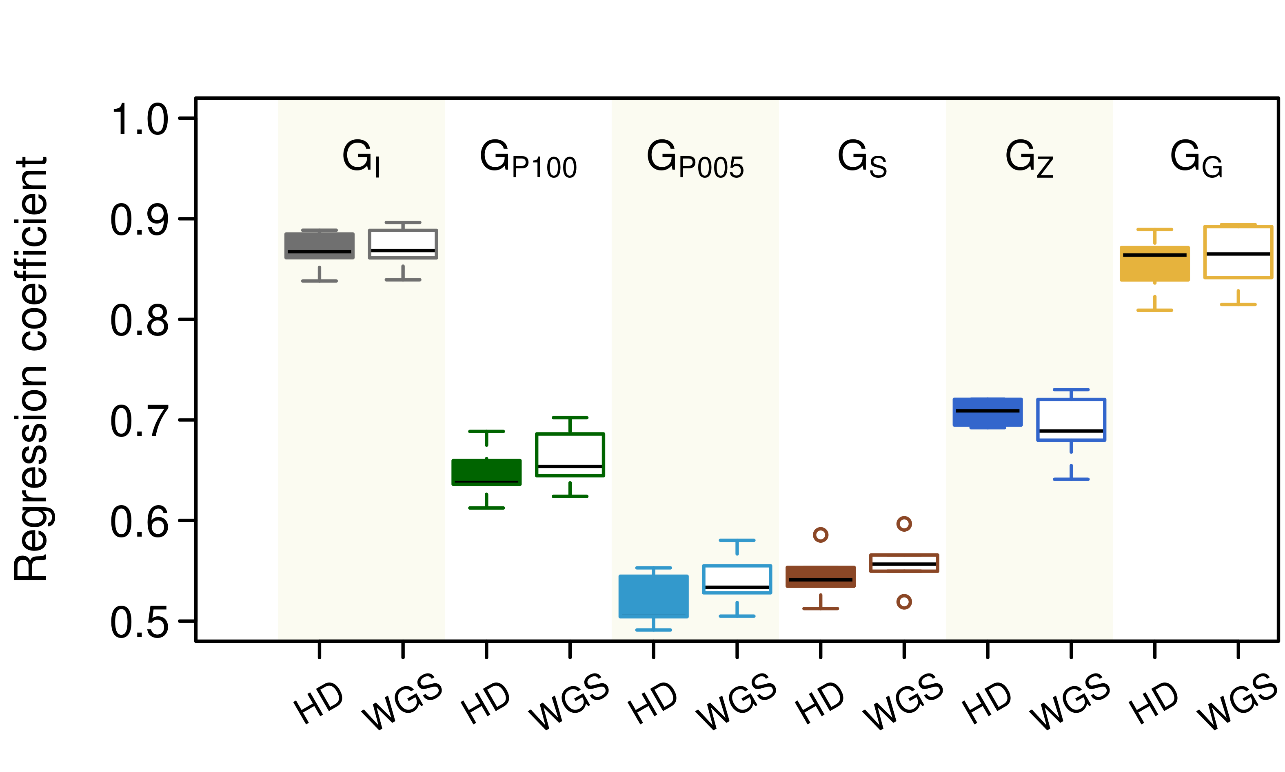

Supplement: Supplementary file 7 — Additional file 7: Figure S4. Regression coefficient of DGV on genomic prediction using different weighting factors based on high-density array data and whole-genome sequencing data. [file 12711_2016_277_MOESM7_ESM.docx]

| 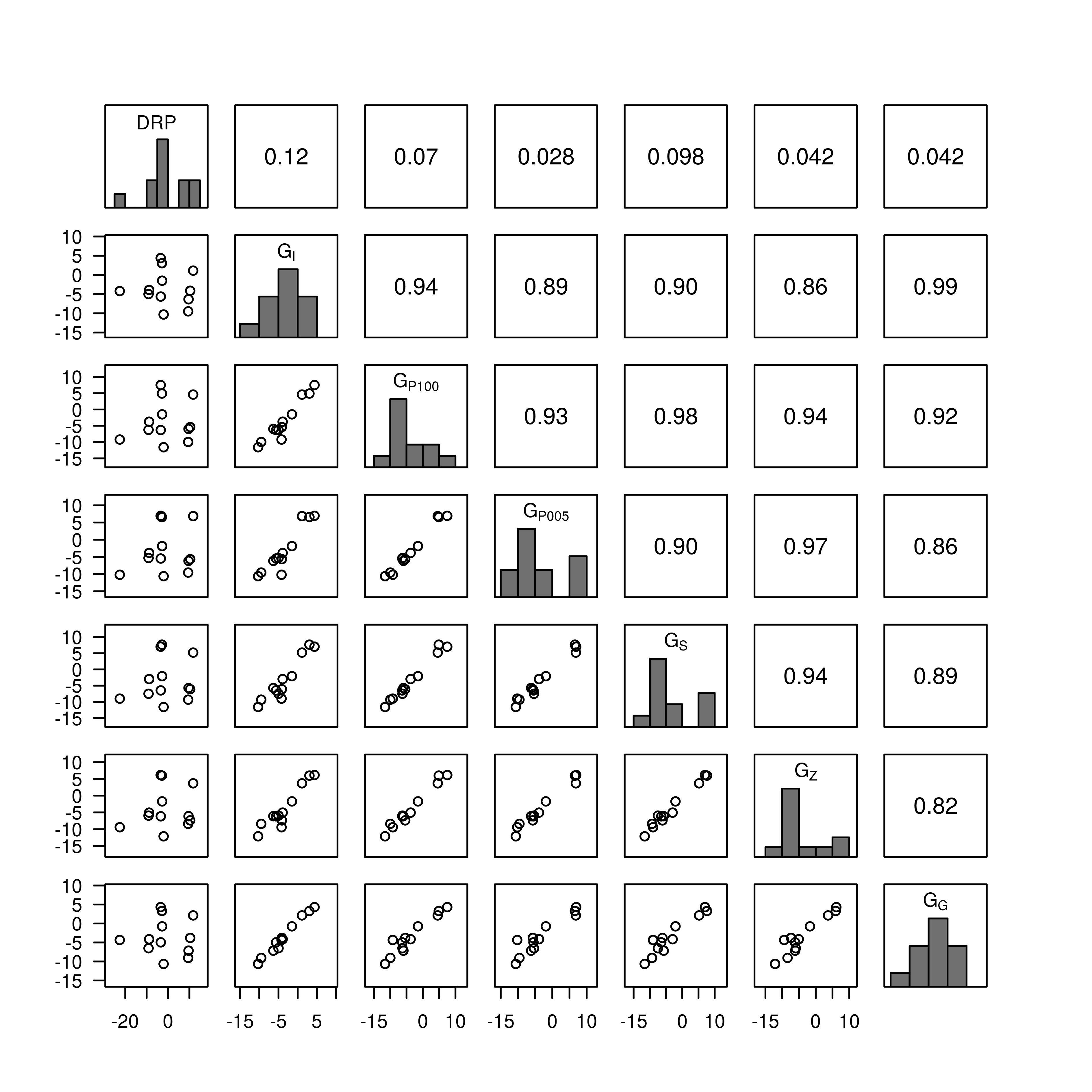 |
| --- |
| 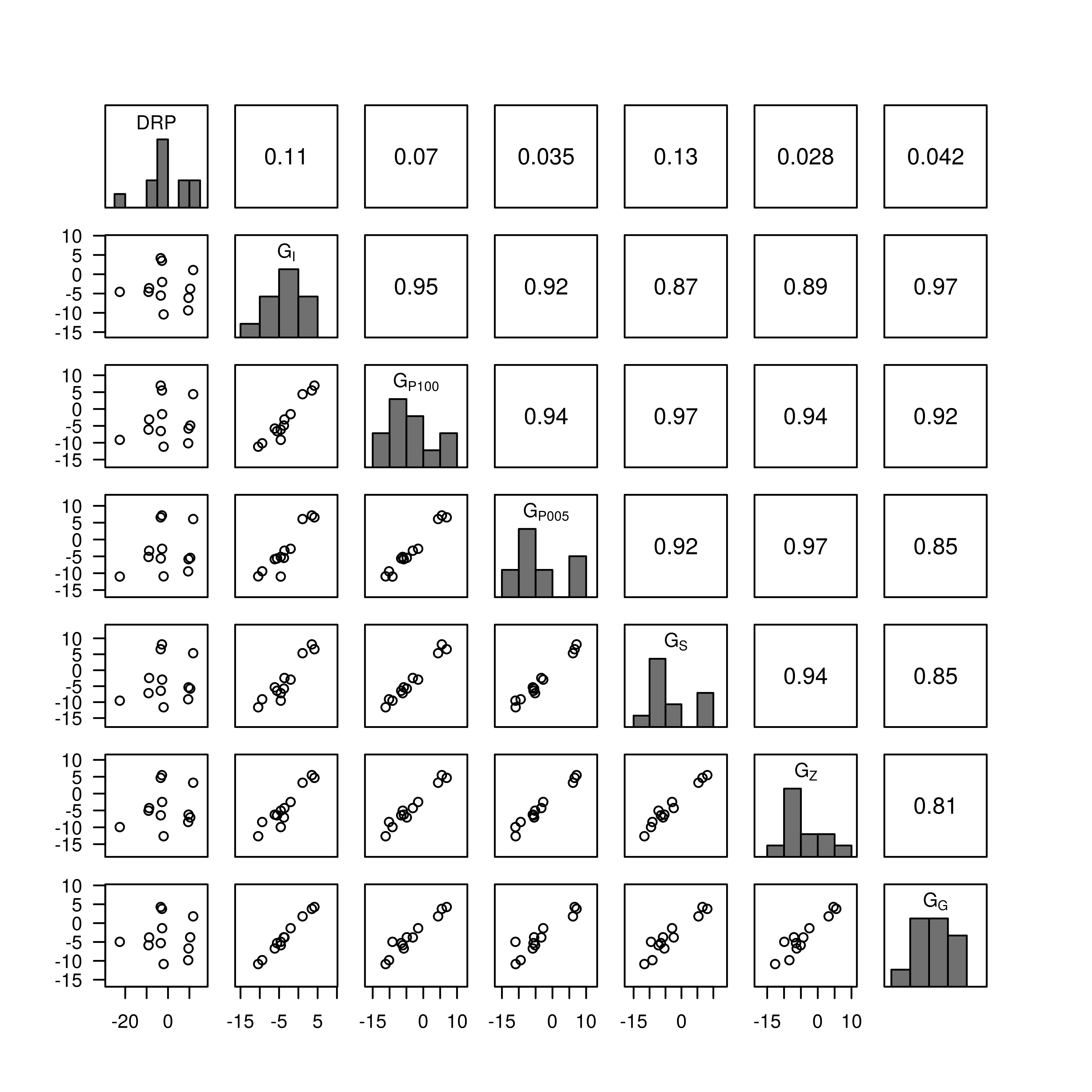 |

Supplement: Supplementary file 8 — Additional file 8: Figure S5. Predictive ability in a full-sib family with 12 individuals for feed intake based on high-density (HD) array data (top) and whole-genome sequence (WGS) data (bottom) of one replicate. In each plot matrix, the diagonal shows the histograms of DRP and DGV obtained with various G matrices. The upper triangle shows the Spearman’s rank correlation between DGV with different G matrices and DRP. The lower triangle shows the scatter plot of DGV with different G matrices and DRP. [file 12711_2016_277_MOESM8_ESM.docx]

| 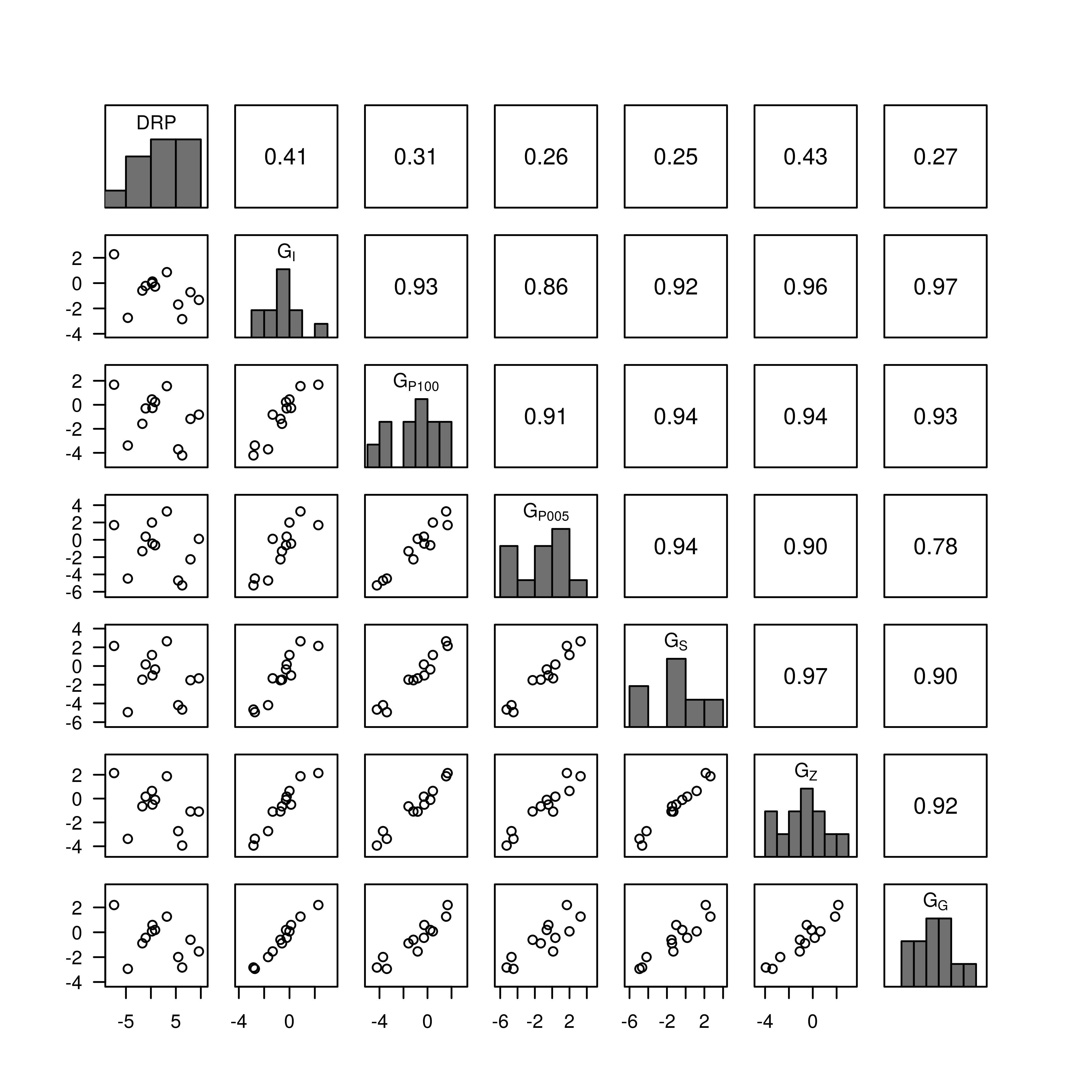 |
| --- |
| 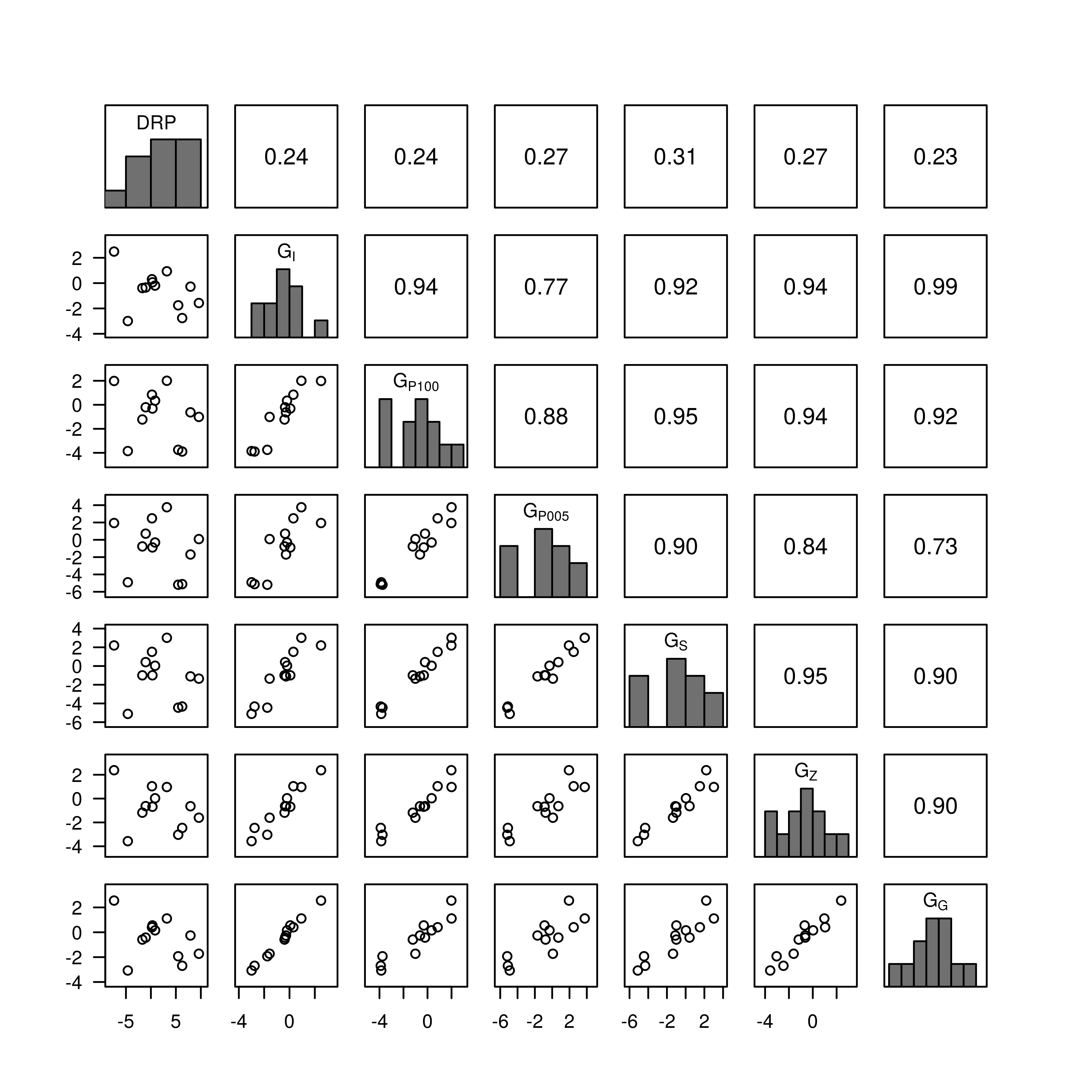 |

Supplement: Supplementary file 9 — Additional file 9: Figure S6. Predictive ability in a full-sib family with 12 individuals for laying rate based on high-density (HD) array data (top) and whole-genome sequence (WGS) data (bottom) of one replicate. In each plot matrix, the diagonal shows the histograms of DRP and DGV obtained with various G matrices. The upper triangle shows the Spearman’s rank correlation between DGV with different G matrices and DRP. The lower triangle shows the scatter plot of DGV with different G matrices and DRP. [file 12711_2016_277_MOESM9_ESM.docx]
