# Supplementary material for: Whole-genome sequence-based genomic prediction in laying chickens with different genomic relationship matrices to account for genetic architecture
Source: Genet Sel Evol. 2017 Jan 16;49:8. doi: 10.1186/s12711-016-0277-y (PMC5238523; doi:10.1186/s12711-016-0277-y)
Supplement: Supplementary file 5 — Additional file 5: Table S2. The optimal parameter in the training stage of BLUP|GA based on HD array data for each fold of fivefold cross-validation in each replicate. [file 12711_2016_277_MOESM5_ESM.docx]

|  |  | Fold1 | | | Fold2 | | | Fold3 | | | Fold4 | | | Fold5 | | |
| --- | --- | --- | --- | --- | --- | --- | --- | --- | --- | --- | --- | --- | --- | --- | --- | --- |
| Trait | rep | top% | $\omega$ | acc | top% | $\omega$ | acc | top% | $\omega$ | acc | top% | $\omega$ | acc | top% | $\omega$ | acc |
| Eggshell  strength | 1 | 10 | 0.1 | 0.368 | 10 | 0.1 | 0.333 | 10 | 0.1 | 0.358 | 10 | 0.1 | 0.373 | 10 | 0.1 | 0.413 |
|  | 2 | 10 | 0.1 | 0.326 | 10 | 0.1 | 0.284 | 10 | 0.1 | 0.360 | 2.5 | 0.1 | 0.373 | 0.2 | 0.1 | 0.413 |
|  | 3 | 1 | 0.1 | 0.395 | 10 | 0.1 | 0.369 | 10 | 0.1 | 0.357 | 10 | 0.1 | 0.361 | 0.1 | 0.1 | 0.367 |
|  | 4 | 1 | 0.1 | 0.393 | 10 | 0.1 | 0.305 | 0.3 | 0.1 | 0.342 | 10 | 0.1 | 0.391 | 10 | 0.1 | 0.378 |
|  | 5 | 10 | 0.1 | 0.379 | 10 | 0.1 | 0.336 | 10 | 0.1 | 0.400 | 10 | 0.1 | 0.329 | 10 | 0.1 | 0.399 |
| Feed  intake | 1 | 10 | 0.2 | 0.399 | 5 | 0.1 | 0.388 | 10 | 0.1 | 0.419 | 5 | 0.2 | 0.394 | 0.4 | 0.1 | 0.375 |
|  | 2 | 10 | 0.1 | 0.427 | 5 | 0.2 | 0.362 | 10 | 0.1 | 0.379 | 10 | 0.7 | 0.413 | 10 | 0.1 | 0.366 |
|  | 3 | 5 | 0.1 | 0.392 | 10 | 0.7 | 0.385 | 5 | 0.4 | 0.352 | 10 | 0.6 | 0.371 | 10 | 0.1 | 0.443 |
|  | 4 | 10 | 0.2 | 0.439 | 10 | 0.1 | 0.369 | 10 | 0.4 | 0.406 | 10 | 0.1 | 0.392 | 5 | 0.2 | 0.450 |
|  | 5 | 10 | 0.5 | 0.379 | 10 | 0.1 | 0.426 | 10 | 0.2 | 0.409 | 2.5 | 0.1 | 0.394 | 0.2 | 0.2 | 0.395 |
| Laying  rate | 1 | 10 | 0.1 | 0.214 | 10 | 0.1 | 0.239 | 10 | 0.1 | 0.212 | 10 | 0.1 | 0.225 | 10 | 0.1 | 0.280 |
|  | 2 | 10 | 0.1 | 0.244 | 10 | 0.1 | 0.239 | 10 | 0.1 | 0.238 | 10 | 0.1 | 0.204 | 10 | 0.1 | 0.196 |
|  | 3 | 10 | 0.1 | 0.181 | 10 | 0.1 | 0.283 | 10 | 0.1 | 0.260 | 10 | 0.1 | 0.230 | 0.05 | 0.1 | 0.184 |
|  | 4 | 10 | 0.1 | 0.183 | 10 | 0.1 | 0.209 | 1 | 0.7 | 0.255 | 10 | 0.1 | 0.235 | 10 | 0.1 | 0.212 |
|  | 5 | 10 | 0.1 | 0.248 | 0.5 | 0.7 | 0.260 | 0.05 | 0.1 | 0.237 | 10 | 0.1 | 0.222 | 5 | 0.1 | 0.292 |
