# Supplementary material for: Whole-genome sequence-based genomic prediction in laying chickens with different genomic relationship matrices to account for genetic architecture
Source: Genet Sel Evol. 2017 Jan 16;49:8. doi: 10.1186/s12711-016-0277-y (PMC5238523; doi:10.1186/s12711-016-0277-y)
Supplement: Supplementary file 6 — Additional file 6: Table S3. The optimal parameter in the training stage of BLUP|GA based on WGS data for each fold of fivefold cross-validation in each replicate. [file 12711_2016_277_MOESM6_ESM.docx]

|  |  | Fold1 | | | Fold2 | | | Fold3 | | | Fold4 | | | Fold5 | | |
| --- | --- | --- | --- | --- | --- | --- | --- | --- | --- | --- | --- | --- | --- | --- | --- | --- |
| Trait | rep | top% | $\omega$ | acc | top% | $\omega$ | acc | top% | $\omega$ | acc | top% | $\omega$ | acc | top% | $\omega$ | acc |
| Eggshell  strength | 1 | 10 | 0.1 | 0.378 | 10 | 0.1 | 0.346 | 0.1 | 0.1 | 0.356 | 10 | 0.1 | 0.382 | 10 | 0.1 | 0.427 |
|  | 2 | 10 | 0.1 | 0.329 | 10 | 0.1 | 0.302 | 10 | 0.1 | 0.367 | 2.5 | 0.1 | 0.386 | 1 | 0.1 | 0.422 |
|  | 3 | 5 | 0.1 | 0.415 | 10 | 0.1 | 0.373 | 10 | 0.1 | 0.367 | 10 | 0.1 | 0.364 | 0.1 | 0.1 | 0.375 |
|  | 4 | 1 | 0.1 | 0.408 | 1 | 0.1 | 0.308 | 10 | 0.1 | 0.350 | 10 | 0.1 | 0.405 | 10 | 0.1 | 0.384 |
|  | 5 | 10 | 0.1 | 0.389 | 10 | 0.1 | 0.340 | 10 | 0.1 | 0.412 | 10 | 0.1 | 0.336 | 10 | 0.1 | 0.407 |
| Feed  intake | 1 | 10 | 0.2 | 0.394 | 10 | 0.1 | 0.394 | 10 | 0.1 | 0.418 | 10 | 0.3 | 0.391 | 0.2 | 0.1 | 0.368 |
|  | 2 | 10 | 0.1 | 0.420 | 10 | 0.3 | 0.356 | 10 | 0.1 | 0.385 | 10 | 0.6 | 0.413 | 10 | 0.1 | 0.372 |
|  | 3 | 10 | 0.1 | 0.395 | 10 | 0.7 | 0.390 | 10 | 0.2 | 0.353 | 10 | 0.6 | 0.375 | 10 | 0.1 | 0.447 |
|  | 4 | 10 | 0.2 | 0.441 | 10 | 0.1 | 0.371 | 10 | 0.3 | 0.409 | 10 | 0.1 | 0.388 | 5 | 0.2 | 0.447 |
|  | 5 | 10 | 0.5 | 0.395 | 10 | 0.1 | 0.434 | 10 | 0.3 | 0.411 | 5 | 0.1 | 0.400 | 0.4 | 0.1 | 0.378 |
| Laying  rate | 1 | 10 | 0.1 | 0.218 | 0.5 | 0.1 | 0.232 | 0.05 | 0.8 | 0.218 | 10 | 0.1 | 0.227 | 10 | 0.1 | 0.285 |
|  | 2 | 0.5 | 0.1 | 0.254 | 10 | 0.1 | 0.240 | 10 | 0.1 | 0.241 | 10 | 0.1 | 0.204 | 10 | 0.1 | 0.204 |
|  | 3 | 10 | 0.1 | 0.178 | 10 | 0.1 | 0.285 | 10 | 0.1 | 0.261 | 10 | 0.1 | 0.229 | 0.1 | 0.99 | 0.189 |
|  | 4 | 10 | 0.1 | 0.180 | 10 | 0.1 | 0.216 | 2.5 | 0.7 | 0.253 | 10 | 0.1 | 0.240 | 2.5 | 0.1 | 0.217 |
|  | 5 | 10 | 0.1 | 0.247 | 0.3 | 0.2 | 0.278 | 10 | 0.1 | 0.236 | 10 | 0.1 | 0.228 | 2.5 | 0.1 | 0.285 |
